# Dioctanoyl Ultrashort Tetrabasic β-Peptides Sensitize Multidrug-Resistant Gram-Negative Bacteria to Novobiocin and Rifampicin

**DOI:** 10.3389/fmicb.2021.803309

**Published:** 2021-12-23

**Authors:** Danyel Ramirez, Liam Berry, Ronald Domalaon, Yanqi Li, Gilbert Arthur, Ayush Kumar, Frank Schweizer

**Affiliations:** ^1^Department of Chemistry, University of Manitoba, Winnipeg, MB, Canada; ^2^Department of Microbiology, University of Manitoba, Winnipeg, MB, Canada; ^3^Department of Biochemistry and Medical Genetics, University of Manitoba, Winnipeg, MB, Canada; ^4^Department of Medical Microbiology and Infectious Diseases, University of Manitoba, Winnipeg, MB, Canada

**Keywords:** antibiotic adjuvant, novobiocin, rifampicin, peptidomimetic, β-amino acid, *Pseudomonas aeruginosa*, *Acinetobacter baumannii*, *Escherichia coli*

## Abstract

Recently reported peptidomimetics with increased resistance to trypsin were shown to sensitize priority multidrug-resistant (MDR) Gram-negative bacteria to novobiocin and rifampicin. To further optimize proteolytic stability, β-amino acid-containing derivatives of these compounds were prepared, resulting in three dioctanoyl ultrashort tetrabasic β-peptides (dUSTBβPs). The nonhemolytic dUSTBβP 3, comprised of three β^3^-homoarginine residues and two fatty acyl tails eight carbons long, enhanced the antibacterial activity of various antibiotics from different classes. Notably, compound 3 retained the ability to potentiate novobiocin and rifampicin in wild-type Gram-negative bacteria against MDR clinical isolates of *Pseudomonas aeruginosa*, *Acinetobacter baumannii*, *Escherichia coli*, *Klebsiella pneumoniae*, and *Enterobacter cloacae*. dUSTBβP 3 reduced the minimum inhibitory concentration of novobiocin and rifampicin below their interpretative susceptibility breakpoints. Furthermore, compound 3 exhibited improved *in vitro* stability (86.8 ± 3.7% remaining) relative to its α-amino acid-based counterpart (39.5 ± 7.4% remaining) after a 2 h incubation in human plasma.

## Introduction

Antimicrobial resistance is a major threat to the global healthcare system that has caused a lack of treatment options for challenging bacterial infections (Prestinaci et al., 2015). The relative decrease in successful antibiotic development in the past decades in addition to the rising transmission of resistance genes has accelerated the problem and new therapies are urgently needed (O’Neill, 2016). Antimicrobial resistance can occur through several distinct mechanisms, including efflux, reduced antibiotic membrane penetration, modification of antibiotic targets, and the production of enzymes to degrade antibiotics (Reygaert, 2018). Of special concern are Gram-negative bacteria due to their highly restrictive outer membrane (OM), which prevents entry of many antibiotics (Zgurskaya and Rybenkov, 2019). Recent advancements have shown the successful development of a number of new antibiotics, as well as increased interest in the use of antibiotic adjuvants to overcome antimicrobial resistance (Årdal et al., 2020).

Adjuvants are molecules that enhance the efficacy of partner antibiotics in combination therapy (Wright, 2016). Adjuvants work primarily by disabling either innate or adaptive bacterial resistance mechanisms. Examples include β-lactamase inhibitors that prevent the enzymatic degradation of β-lactam antibiotics (Drawz and Bonomo, 2010), bacterial efflux pump inhibitors (Lamers et al., 2013), and OM-permeabilizing agents derived from cationic antimicrobial peptides (AMPs). Currently, the only adjuvants approved for clinical use are β-lactamase inhibitors, including clavulanic acid (combined with amoxicillin), vaborbactam (combined with meropenem), and others (Carcione et al., 2021). Membrane-permeabilizing adjuvants derived from the polymyxin family of AMPs have also seen some pre-clinical success. One example is polymyxin B nonapeptide (PMBN), which lacks the fatty acyl tail and the Dab_1_ amino acid characteristic of polymyxins (Ofek et al., 1994). Despite a reduction in antibacterial activity, PMBN is able to permeabilize the OM of Gram-negative bacteria and allow other antibiotics to enter the cell at increased rates (Vaara, 1992). Spero Therapeutics is also presently developing SPR741, an adjuvant derived from PMBN, which has reduced toxicity and improved pharmacokinetics (Eckburg et al., 2019; Vaara, 2019). SPR741 was assessed for safety, tolerability, and in combination with partner antibiotics in Phase 1 clinical trials (Eckburg et al., 2019; Vaara, 2019). Various short cationic lipopeptide adjuvants have also been reported that display similar membrane permeabilizing properties (Domalaon et al., 2018a,b, 2019a; Ramirez et al., 2020).

A common feature of the activity of AMPs against Gram-negative bacteria is the presence of basic sidechains, which can be protonated at physiological pH (Velkov et al., 2010). These confer an overall positive charge to the peptide, which can electrostatically interact with negatively charged phosphate groups embedded in the lipid A component of the lipopolysaccharide of the OM. This interaction results in the displacement of divalent cations such as Ca^2+^ and Mg^2+^ which normally stabilize the negatively charged lipopolysaccharide (Hancock, 1984). After OM integrity has been disrupted, the fatty acyl tail of polymyxin inserts into the phospholipid bilayer causing lipid rearrangement that consequently results in cell lysis and cell death (Moubareck, 2020). Although PMBN lacks this fatty acyl tail and thus is unable to lyse the cell, localized perturbation of the membrane still occurs (Tsubery et al., 2000). Despite their potent activity as both antibiotics and adjuvants (Melander and Melander, 2017; Hollmann et al., 2018; Marquette and Bechinger, 2018; Sheard et al., 2019; Vaara, 2019), AMPs face significant challenges toward clinical use. Notable challenges include high production cost, relatively poor metabolic stability, and overall high toxicity due to hemolysis and nephrotoxicity (Chen and Lu, 2020).

**Figure 1:**
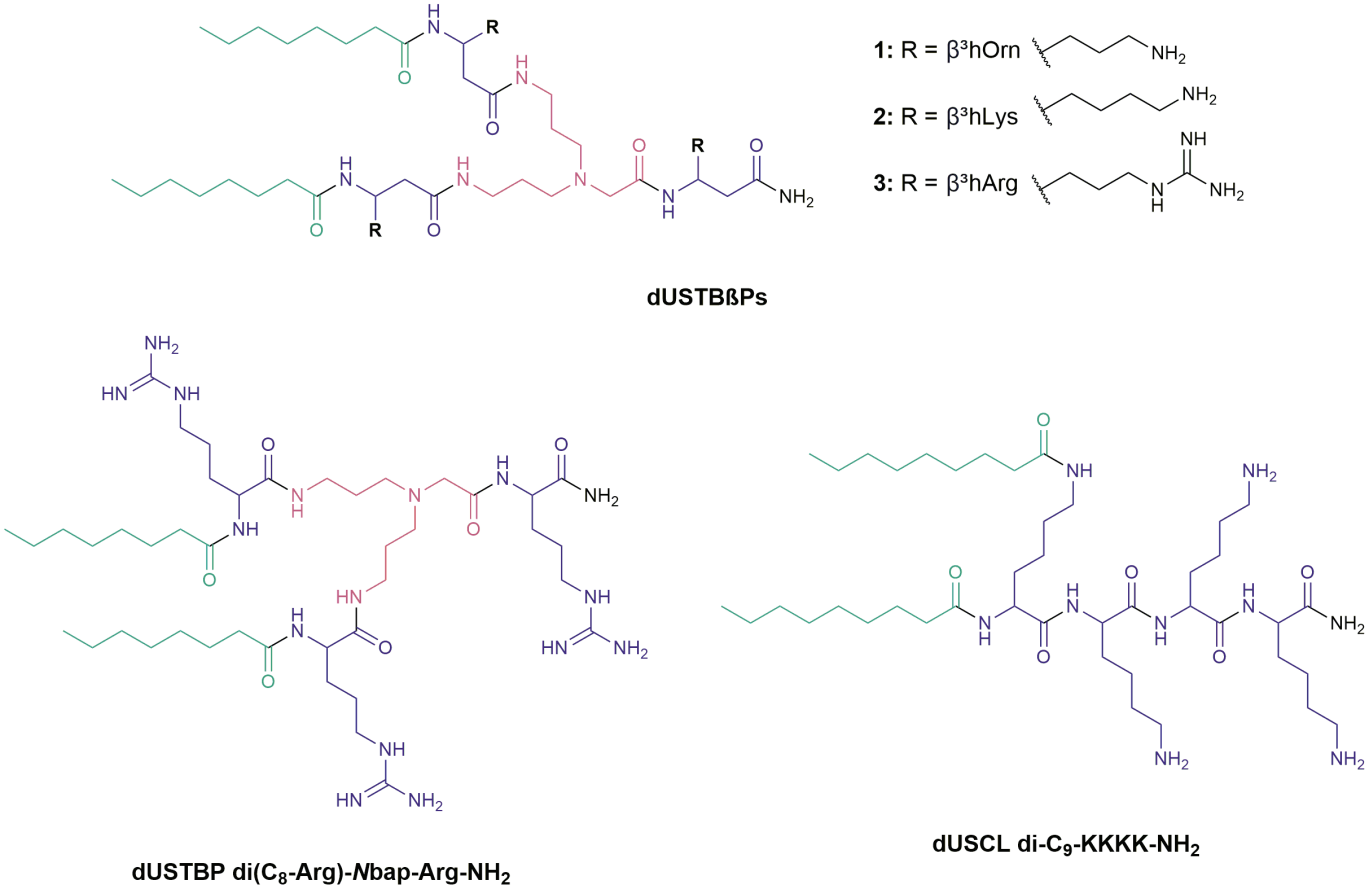
Chemical structures of newly synthesized dioctanoyl ultrashort tetrabasic β-peptides (dUSTBβPs) and reference compounds.

Various approaches to improve the drug-likeness of AMPs have been reported. For instance, the above-mentioned PMBN and SPR741 have reduced toxicity (Vaara, 2019). We have previously reported dilipid ultrashort cationic lipopeptides (dUSCLs) containing two shorter instead of one longer fatty acyl tail that resulted to reduced hemolysis (Domalaon et al., 2019a). Metabolic instability due to protease activity remains a significant challenge for AMPs. Peptidomimetic approaches can mitigate proteolytic susceptibility by removing the peptide-like character of AMPs thereby resulting in the inability of proteases to recognize and degrade the resulting mimic molecule (Domalaon et al., 2016). Several peptidomimetic strategies can be enacted in lead AMP candidates including isosteric replacement of the peptide backbone, replacing naturally occurring L-amino acids with D-amino acids, and the use of β-amino acids and peptoid building blocks (Rink et al., 2010; Molchanova et al., 2017; Baker et al., 2019; Mood et al., 2021). For instance, dilipid ultrashort tetrabasic peptidomimetics (dUSTBPs) were previously developed from dUSCL lead candidates by introducing the branched molecular scaffold, *N*,*N*-bis(3-aminopropyl)glycine (*N*bap), into the structure to interrupt the peptide backbone (Ramirez et al., 2020). The nonhemolytic dUSTBP di(C_8_-Arg)-*N*bap-Arg-NH_2_ (Figure 1) enhanced the antibacterial activity of novobiocin and rifampicin, and was shown to have increased resistance to trypsin relative to dUSCL di-C_9_-KKKK-NH_2_ (Figure 1; Ramirez et al., 2020). Although improved peptide stability was observed in dUSTBPs, our continued effort in optimizing our lead candidates by incorporating further peptidomimetic structural features is reported herein.

Replacement of traditional α-amino acids in dUSTBPs with β-amino acid analogues resulted in dioctanoyl ultrashort tetrabasic β-peptides (dUSTBβPs). Three dUSTBβPs were prepared by incorporating fatty acids eight carbons (C_8_) long and β-amino acids including β^3^-homoornithine (β^3^hOrn), β^3^-homolysine (β^3^hLys), or β^3^-homoarginine (β^3^hArg; Figure 1). The dUSTBβPs were all nonhemolytic and potentiated novobiocin and rifampicin against wild-type Gram-negative bacteria. Peptidomimetic 3, consisting of three β^3^hArg residues, enhanced the antibacterial activity of various antibiotics from different classes, as well as retained novobiocin and rifampicin potentiation against multidrug-resistant (MDR) clinical isolates. Moreover, compound 3 was shown to possess enhanced plasma stability relative to its α-amino acid-based counterpart di(C_8_-Arg)-*N*bap-Arg-NH_2_. These results indicate that multiple peptidomimetic approaches can serve to further improve proteolytic resistance of AMPs without compromising adjuvant activity.

## Materials and Methods

### Preparation of dUSTBβPs

The Rink amide 4-methylbenzyhydrylamine (MBHA) resin was obtained from Sigma-Aldrich (United States), Fmoc-β^3^-hOrn(Boc)-OH was obtained from A2B Chem (United States), and Fmoc-β^3^-hArg(Pbf)-OH was obtained from 1Click Chemistry (United States). Fmoc-β^3^-hLys(Boc)-OH and *N*,*N*-bis(N′-Fmoc-3-aminopropyl)glycine potassium hemisulfate were purchased from Chem-Impex (United States). All other reagents and solvents were obtained from Sigma-Aldrich (United States) and used without further purification.

All dUSTBβPs were prepared by following a standard fluorenylmethyloxycarbonyl (Fmoc) solid-phase peptide synthesis (SPPS) protocol using Rink amide MBHA resin (Chan and White, 2000). N-terminus of the amino acids was protected with Fmoc. The *ϖ-* and *ω-*amine sidechains of β^3^hOrn and β^3^hLys, and the *ω-*amine sidechain of β^3^hArg were protected with *tert*-butyloxycarbonyl and 2,2,4,6,7-pentamethyldihydrobenzofuran-5-sulfonyl, respectively. Fmoc deprotection was carried out using 20% piperidine in dimethylformamide (DMF; v/v). Peptide and fatty acid coupling were done *via* addition of a preactivated coupling solution to the resin and subsequent constant gentle agitation with nitrogen gas for 45 min. The coupling solution which consists of 3 molar equivalents (mol. eq.) of protected amino acid or lipid, 3 mol. eq. of *O-*(benzotriazol-1-yl)-*N*,*N*,*N′*,*N′*-tetramethyluronium tetrafluoroborate, and 8 mol. eq. of *N-*methylmorpholine in DMF was preactivated for 5 min. Following both Fmoc deprotection and coupling steps, the resin was washed with DMF (3×), dichloromethane (DCM; 3×), and DMF (3×) to remove traces of piperidine from the reaction vessel. The chloranil test (2% chloranil in DMF) was performed on a small amount of resin to verify the completion of the reaction. Deprotection of the amino acid sidechains and cleavage of the peptide from the resin were done using 95:5 trifluoroacetic acid (TFA)/water (v/v) for 30 min. The resin was washed with DCM (3×), and the solvent was removed *in vacuo* to afford the solid compounds as TFA salts. Molecular weights of the dUSTBβPs in TFA salt form are shown in Supplementary Table 1.

The dUSTBβPs were purified *via* reverse-phase flash chromatography using C_18_ silica gel (40–63 μm) purchased from Silicycle (United States). The solvent system used consisted of methanol and water containing 0.1% TFA. Purity of the compounds was determined to be ≥95% using high-performance liquid chromatography (HPLC) on a Thermo Scientific Vanquish UHPLC (United States) equipped with Phenomenex Kinetex (100 mm × 4.6 mm) 2.6 μm XB-C18 reverse-phase column and VF-D40 variable wavelength detector. The HPLC gradient used for purity analysis is shown in Supplementary Table 8. Chemical characterization of each dUSTBβP was assessed by one- and two-dimensional nuclear magnetic resonance experiments, such as ^1^H, ^13^C, COSY, HSQC, and HMBC on a Bruker AMX-500 (500 MHz) instrument (Germany). Matrix-assisted laser desorption ionization-time of flight mass spectrometry experiments were carried out on a Bruker Ultraflextreme (Germany) in positive ion mode with 2,5-dihydroxybenzoic acid as the matrix.

### Bacterial Strains and Growth Conditions

Bacterial isolates were obtained from the American Type Culture Collection (ATCC), the Canadian National Intensive Care Unit (CAN-ICU) surveillance study (Zhanel et al., 2008), and the Canadian Ward (CAN-WARD) surveillance study (Hoban and Zhanel, 2013). CAN-ICU and CAN-WARD bacterial isolates were collected from patients diagnosed with presumed infectious diseases admitted in participating medical centers across Canada. All pharmaceutical-grade antibiotics and reagents were purchased from commercial sources.

### Hemolysis Assay

The degree of hemolysis induced by dUSTBβPs was determined by the amount of hemoglobin released from human erythrocytes upon incubation. Fresh human blood supplied by a commercial vendor was obtained from normal healthy volunteers following informed consent. The agent of interest was serially diluted in vehicle consisting of phosphate-buffered saline (PBS), saline, 5% glucose, or equivalents. The samples were incubated with gentle mixing at 37°C for 45 min and were subsequently centrifuged. The supernatant (plasma layer) was removed and centrifuged once more to completely pellet the cells. The plasma layer was diluted with Drabkin’s reagent and analyzed at a wavelength of 540 nm. Experiments were conducted in triplicates, and a calibration curve prepared by diluting blood (after addition of vehicle) was used to quantitate heme release. The vehicle or Triton X-100 were used as negative or positive controls, respectively.

### Antimicrobial Susceptibility Assay

The *in vitro* antibacterial activities of dUSTBβPs were evaluated using microbroth dilution susceptibility test, in accordance with the Clinical and Laboratory Standards Institute (CLSI) guidelines (Clinical and Laboratory Standards Institute, 2019). Overnight-grown bacterial culture diluted in saline to 0.5 McFarland turbidity was successively diluted 1:50 in Mueller-Hinton broth (MHB) to achieve a final concentration of 5 × 10^5^ colony forming units (CFU)/mL for inoculation. The agents were serially diluted 2-fold in MHB on a 96-well plate; mixed with bacterial inoculum of equal volumes, and incubated at 37°C for 18 h. Antibacterial activity was determined by the minimum inhibitory concentration (MIC), which is the lowest concentration of agent required for inhibition of visible bacterial growth in the form of turbidity. Confirmation of turbidity was done using an EMax Plus microplate reader (Molecular Devices, United States) at a wavelength of 590 nm. Wells comprising MHB alone or MHB inoculated with bacteria were used as negative or positive controls, respectively.

### Checkerboard Assay

The adjuvant activities of dUSTBβPs were evaluated using checkerboard assay as previously described (Berry et al., 2019). Overnight-grown bacterial culture diluted in saline to 0.5 McFarland turbidity was successively diluted 1:50 in MHB to achieve a final concentration of 5 × 10^5^ CFU/mL for inoculation. The antibiotic and adjuvant were serially diluted 2-fold along the *x*-axis and *y*-axis, respectively, resulting in varying concentrations of both agents in each well. Subsequently, the 96-well plate was incubated with equal volumes of bacterial inoculum at 37°C for 18 h. Confirmation of turbidity was done using an EMax Plus microplate reader (Molecular Devices, United States) at a wavelength of 590 nm. Wells comprising MHB alone or in the presence of bacterial cells were used as negative or positive controls, respectively. Fractional inhibitory concentration index (FICI) is determined by adding the FICs of both antibiotic and adjuvant. The FIC of the antibiotic is calculated by dividing the MIC of the antibiotic in the presence of the adjuvant by the MIC of the antibiotic alone. Similarly, the FIC of the adjuvant is calculated by dividing the MIC of the adjuvant in the presence of the antibiotic by the MIC of the adjuvant alone. FICI ≤ 0.5, 0.5 < *x* ≤ 4, and >4 were deemed synergistic, additive, and antagonistic, respectively (Meletiadis et al., 2010).

### Time-Kill Assay

The concentration-dependent killing kinetics of the combinations of dUSTBβP 3 and novobiocin or rifampicin was studied using time-kill assay as previously described (Domalaon et al., 2019b). Overnight-grown bacterial culture diluted in PBS to 0.5 McFarland turbidity was successively diluted 1:50 in lysogeny broth (LB). Cell cultures in the presence of dUSTBβP 3, novobiocin, or rifampicin, or combinations of adjuvant and antibiotic were incubated at 37°C. At designated intervals, 100 μL aliquots acquired from each culture tube were serially diluted in PBS and plated on LB agar plates. After incubation of the plates at 37°C for 18 h, the bacterial colonies were counted.

### OM Permeabilization Assay

The ability of dUSTBβP 3 to permeabilize the OM of *Acinetobacter baumannii* ATCC 17978 and *Escherichia coli* ATCC 25922 was assessed using 1-*N*-phenylnaphthylamine (NPN) as previously described with minor modifications (Yang et al., 2017; Akhoundsadegh et al., 2019). Overnight grown culture was subcultured (1 in 100) in fresh LB broth and grown to a mid-logarithmic phase (OD_600_ = 0.4–0.6). The cells were pelleted by centrifugation for 10 min at 1,200 × *g* at room temperature, washed, and resuspended in half volume of 5 mM 4-(2-hydroxyethyl)-1-piperazineethanesulfonic acid (HEPES; pH 7.2) with 5 mM glucose. NPN (10 μM final concentration) was added to a black 96-well plate containing the cell culture and incubated in 5 mM HEPES (pH 7.2) supplemented with 5 mM glucose and 5 μM carbonyl cyanide 3-chlorophenylhydrazone at room temperature for 30 min in darkness. Varying concentrations of compound were added onto the suspension, and the resulting change in NPN fluorescence was measured continuously (every 30 s) on a SpectraMax M2 microplate reader (Molecular Devices, United States) at an excitation wavelength of 350 nm and an emission wavelength of 420 nm. Cells with NPN and the OM permeabilizer PMBN served as a positive control, while cells with NPN alone served as a negative control. Three replicates were conducted, and the data were corrected for any background fluorescence.

### Tryptic Digest Assay

Proteolytic resistance of dUSTBβP 3 and previously reported dUSCL di-C_9_-KKKK-NH_2_ was evaluated with tryptic digest assay as previously described (Ramirez et al., 2020). The compounds were diluted with 50 mM ammonium bicarbonate (pH 7.8) and were incubated with sequencing-grade modified trypsin from Promega (United States) at a molar ratio of 1:5,000 (enzyme/compound) at 37°C for 2 h. Termination of the reaction was done by overnight freezing at −18°C, and the samples were purified and concentrated by using Pierce C_18_ tips (10 μL) from Thermo Scientific (United States). Stability toward trypsin was assessed by mass fragmentation analysis in positive ion mode on a Varian 500-MS ion trap mass spectrometer (United States).

### Plasma Stability Assay

Stability of dUSTBβP 3 was evaluated in human plasma. Compound 3 was incubated at 37°C in prewarmed plasma for a final compound concentration of 1 μM. At designated time points (0, 0.5, 1, 1.5, and 2 h), aliquots of the mixture were diluted with acetonitrile, and centrifuged at 1,000 × *g* for 15 min at 4°C. The supernatant was analyzed by HPLC-tandem mass spectrometry using selected reaction monitoring. The % compound remaining was determined by comparing peak areas at different time points to time zero. Assuming first-order kinetics, the half-life was extrapolated from the slope of the initial linear range of the logarithmic curve of compound remaining over time. Experiments were performed in duplicates, and the reference compounds include propantheline and propoxycaine.

### Cell Viability Assay

Cell viability assay was performed essentially as previously described (Ammeter et al., 2019). Human embryonic kidney cells (HEK293) and human liver carcinoma cells (HepG2) were grown in Dulbecco’s modified eagle’s medium with 10% fetal bovine serum at 37°C using a humidified 5% CO_2_ incubator in 75 mm tissue culture flasks. The cells were detached with trypsin and equal numbers of cells (8,000 cells in 50 μL) were dispersed into five rows of each column in 96-well plate; the remaining three rows of each column, designated as blanks, received 50 μL of the media without cells. After 24 h incubation, aliquots of 50 μL of dUSTBβP 3 at varying concentrations were added to each well and incubated for 48 h. Thereafter, cell viability was measured using PrestoBlue Cell Viability reagent from Invitrogen (United States) according to manufacturer’s protocol and fluorescence (540/590 nm) was measured with a SpectraMax M2 plate reader (Molecular Devices, United States). Values from the wells without cells (blank wells) were subtracted from the corresponding sample wells and cell viability values of the treated samples relative to the vehicle controls set to 100% was determined. Thus, relative cell viability of 0% indicates that there are no viable cells. The results represent the mean ± SD of two independent experiments with five samples per experiment. The concentration that causes 50% cytotoxicity (CC_50_) was also estimated by using non-linear regression analysis. Colistin was used as a negative control and doxorubicin, an anticancer drug, was used as a positive control.

## Results

### Hemolytic and Susceptibility Screening of dUSTBβPs

**Figure 2:**
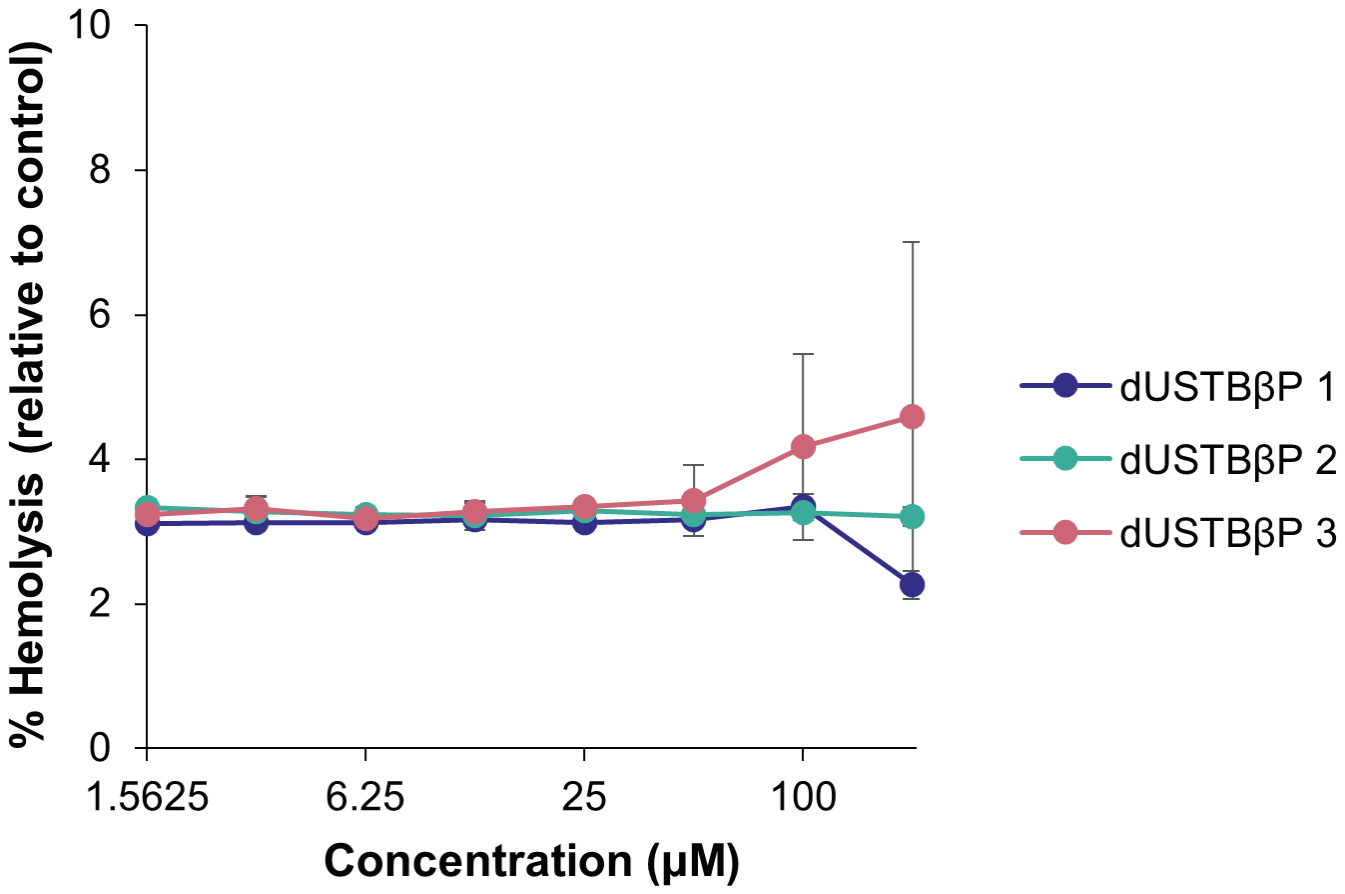
Concentration-dependent hemolytic activity of dUSTBβPs against human erythrocytes. Control used was 1% Triton X-100. Results were an average of triplicates (*n* = 3) ± SD. See Supplementary Tables 2, 3 for exact values of % hemolysis and SDs.

The ability of the compounds to cause lysis of red blood cells was determined by measuring the amount of hemoglobin released in plasma upon treatment (Figure 2). The β-amino acid-containing derivatives elicited low levels of hemolysis at all concentrations examined. At 200 μM, compounds 1, 2, and 3 only resulted in 2.26 ± 0.19, 3.21 ± 0.13, and 4.59 ± 2.42% hemolysis, respectively. To examine the susceptibility of wild-type Gram-negative bacteria to dUSTBβPs, MICs of the synthesized compounds were determined (Table 1). Limited activity of ≥128 μg/mL was observed for all the compounds against all tested strains.

**Table 1:** Antibacterial activity of dUSTBβPs against wild-type Gram-negative bacteria.

Organism	MIC (μg/mL)			
	1	2	3	*P*. *aeruginosa* PAO1
>128	>128	128	*A. baumannii* ATCC 17978	>128
>128	>128	*E*. *coli* ATCC 25922	>128	>128
128				

### dUSTBβPs Potentiated Novobiocin and Rifampicin Against Wild-Type Gram-Negative Bacteria

The capability of dUSTBβPs to potentiate novobiocin and rifampicin was assessed against wild-type *Pseudomonas aeruginosa* (Table 2), *A. baumannii* (Table 3), and *E. coli* (Table 4) by means of a checkerboard assay. The FICI was used to evaluate interactions between the two agents. FICI of ≤0.5, 0.5 < *x* ≤ 4, and >4 were interpreted as synergy, additive, and antagonistic, respectively (Meletiadis et al., 2010). All interactions of dUSTBβPs with novobiocin and rifampicin were synergistic against all tested strains. The combinations were most effective against *E. coli* (FICI of 0.016–0.125) and *A. baumannii* (FICI of 0.004–0.188), and least effective against PAO1 (FICI of 0.078–0.375).

**Table 2:** Synergy evaluation of combinations consisting of dUSTBβPs and novobiocin or rifampicin against wild-type *P. aeruginosa* PAO1.

dUSTBβP	Antibiotic	MIC_dUSTBβP_ [MIC_combo_] (μg/mL)	MIC_antibiotic_ [MIC_combo_] (μg/mL)	FICI	Interpretation	Absolute MIC^a^_antibiotic_ (μg/mL)	Potentiation^b^
1	Novobiocin	>128 [8]	1,024 [128]	0.125 < *x* < 0.188	Synergy	128	8-fold
	Rifampicin	>128 [16]	16 [4]	0.25 < *x* < 0.375	Synergy	8	2-fold
2	Novobiocin	>128 [8]	1,024 [128]	0.125 < *x* < 0.188	Synergy	128	8-fold
	Rifampicin	>128 [16]	16 [4]	0.25 < *x* < 0.375	Synergy	8	2-fold
3	Novobiocin	128 [8]	1,024 [32]	0.094	Synergy	32	32-fold
	Rifampicin	128 [8]	16 [0.25]	0.078	Synergy	0.25	64-fold

a MIC of antibiotic in the presence of 8 μg/mL (6 μM) dUSTBβP.

b Degree of antibiotic potentiation in the presence 8 μg/mL (6 μM) dUSTBβP.

**Table 3:** Synergy evaluation of combinations consisting of dUSTBβPs and novobiocin or rifampicin against wild-type *A. baumannii* ATCC 17978.

dUSTBβP	Antibiotic	MIC_dUSTBβP_ [MIC_combo_] (μg/mL)	MIC_antibiotic_ [MIC_combo_] (μg/mL)	FICI	Interpretation	Absolute MIC^a^_antibiotic_ (μg/mL)	Potentiation^b^
1	Novobiocin	>128 [8]	16 [2]	0.125 < *x* < 0.188	Synergy	2	8-fold
	Rifampicin	>128 [8]	2 [0.125]	0.063 < *x* < 0.125	Synergy	0.125	16-fold
2	Novobiocin	>128 [8]	16 [2]	0.125 < *x* < 0.188	Synergy	2	8-fold
	Rifampicin	>128 [8]	2 [0.125]	0.063 < *x* < 0.125	Synergy	0.125	16-fold
3	Novobiocin	>128 [8]	16 [0.25]	0.016 < *x* < 0.078	Synergy	0.25	64-fold
	Rifampicin	>128 [8]	2 [0.008]	0.004 < *x* < 0.066	Synergy	0.008	256-fold

a MIC of antibiotic in the presence of 8 μg/mL (6 μM) dUSTBβP.

b Degree of antibiotic potentiation in the presence 8 μg/mL (6 μM) dUSTBβP.

**Table 4:** Synergy evaluation of combinations consisting of dUSTBβPs and novobiocin or rifampicin against wild-type *E. coli* ATCC 25922.

dUSTBβP	Antibiotic	MIC_dUSTBβP_ [MIC_combo_] (μg/mL)	MIC_antibiotic_ [MIC_combo_] (μg/mL)	FICI	Interpretation	Absolute MIC^a^_antibiotic_ (μg/m1)	Potentiation^b^
1	Novobiocin	>128 [4]	64 [2]	0.031 < *x* < 0.063	Synergy	2	32-fold
	Rifampicin	>128 [8]	4 [0.25]	0.063 < *x* < 0.125	Synergy	0.25	16-fold
2	Novobiocin	>128 [8]	64 [1]	0.016 < *x* < 0.078	Synergy	1	64-fold
	Rifampicin	>128 [8]	4 [0.125]	0.031 < *x* < 0.094	Synergy	0.125	32-fold
3	Novobiocin	128 [8]	64 [0.125]	0.064	Synergy	0.125	512-fold
	Rifampicin	128 [8]	4 [0.008]	0.064	Synergy	0.008	512-fold

a MIC of antibiotic in the presence of 8 μg/mL (6 μM) dUSTBβP.

b Degree of antibiotic potentiation in the presence 8 μg/mL (6 μM) dUSTBβP.

### dUSTBβP 3 Retains Novobiocin and Rifampicin Potentiation Against MDR Gram-Negative Bacteria

Potentiation of novobiocin (Table 5) and rifampicin (Table 6) by dUSTBβP 3 was also examined against MDR clinical isolates of *P. aeruginosa*, *A. baumannii*, and *Enterobacteriaceae*. Supplementary Tables 4-6 contain the full data. MDR is characterized by nonsusceptibility to at least one agent in ≥3 different antibiotic categories (Magiorakos et al., 2012). The antibiotic susceptibility profile of the tested strains can be found in Supplementary Table 9. Testing of the α-amino acid-based counterpart di(C_8_-Arg)-*N*bap-Arg-NH_2_ (Figure 1) was included for comparison. Compound 3 synergized with both antibiotics against all clinical isolates tested. Similar to the trend found for the dUSTBP, the combinations of 8 μg/mL (6 μM) compound 3 and novobiocin or rifampicin were most potent against *A. baumannii* (absolute MIC values of 0.002–0.125 μg/mL) and *Enterobacteriaceae* (absolute MIC values of 0.004–32 μg/mL). Moreover, novobiocin or rifampicin in the presence of dUSTBβP 3 was least active against *P. aeruginosa* (absolute MIC values of 0.5–1,024 μg/mL).

**Table 5:** Potentiation of novobiocin by dUSTBP di(C_8_-Arg)-*N*bap-Arg-NH_2_ or dUSTBβP 3 at a fixed concentration of 8 μg/mL (6 μM) against MDR *P. aeruginosa*, *A. baumannii*, and *Enterobacteriaceae*.

Organism	MIC_novobiocin_ (μg/mL)		
	Alone	+ di(C_8_-Arg)-*N*bap-Arg-NH_2_^a^	+ dUSTBβP 3^a^
*P. aeruginosa* PA259-96196	1,024	16	32
*P. aeruginosa* PA262-101856	1,024	32	1,024
*P. aeruginosa* PA264-104354	1,024	4	512
*P. aeruginosa* PA91433	1,024	32	1,024
*P. aeruginosa* PA114228	1,024	256	256
*A. baumannii* AB027	8	0.031	0.063
*A. baumannii* AB031	4	0.031	0.031
*A. baumannii* LAC-4	1	0.008	0.002
*A. baumannii* 92247	4	0.063	0.063
*A. baumannii* 110193	128	0.25	0.125
*E. coli* 94393	64	0.5	0.125
*E. coli* 94474	256	0.5	0.5
*E. coli* 107115	128	0.25	0.5
*K. pneumoniae* 113250	128	2	2
*K. pneumoniae* 113254	256	2	4
*K. pneumoniae* 116381	256	1	1
*E. cloacae* 117029	512	0.5	0.25
*E. cloacae* 118564	256	0.5	0.5
*E. cloacae* 121187	32	2	2

a MIC of novobiocin in the presence of 8 μg/mL (6 μM) compound. MIC of dUSTBP di(C_8_-Arg)-Nbap-Arg-NH_2_ and dUSTBβP 3 is ≥64 μg/mL against all strains tested, with the exception of *A. baumannii* LAC-4 (MIC of 16 μg/mL).

**Table 6:** Potentiation of rifampicin by dUSTBP di(C_8_-Arg)-*N*bap-Arg-NH_2_ or dUSTBβP 3 at a fixed concentration of 8 μg/mL (6 μM) against MDR *P. aeruginosa*, *A. baumannii*, and *Enterobacteriaceae*.

Organism	MIC_rifampicin_ (μg/mL)		
	Alone	+ di(C_8_-Arg)-*N*bap-Arg-NH_2_^a^	+ dUSTBβP 3^a^
*P. aeruginosa* PA259-96196	16	0.5	0.5
*P. aeruginosa* PA262-101856	1,024	32	64
*P. aeruginosa* PA264-104354	16	0.063	0.5
*P. aeruginosa* PA91433	16	1	8
*P. aeruginosa* PA114228	16	2	8
*A. baumannii* AB027	1	0.031	0.008
*A. baumannii* AB031	1	0.016	0.002
*A. baumannii* LAC-4	0.5	0.016	0.004
*A. baumannii* 92,247	2	0.031	0.016
*A. baumannii* 110,193	1	0.031	0.008
*E. coli* 94393	8	0.031	0.016
*E. coli* 94474	8	0.031	0.008
*E. coli* 107115	32	0.002	0.004
*K. pneumoniae* 113250	32	0.5	1
*K. pneumoniae* 113254	16	2	0.5
*K. pneumoniae* 116381	512	32	32
*E. cloacae* 117029	8	0.016	0.004
*E. cloacae* 118564	8	0.25	0.125
*E. cloacae* 121187	4	2	0.25

a MIC of rifampicin in the presence of 8 μg/mL (6 μM) compound. MIC of dUSTBP di(C_8_-Arg)-Nbap-Arg-NH_2_ and dUSTBβP 3 is ≥64 μg/mL against all strains tested, with the exception of *A. baumannii* LAC-4 (MIC of 16 μg/mL).

### Time-Kill Kinetics of dUSTBβP 3 and Novobiocin or Rifampicin in Wild-Type and MDR *A. baumannii*

**Figure 3:**
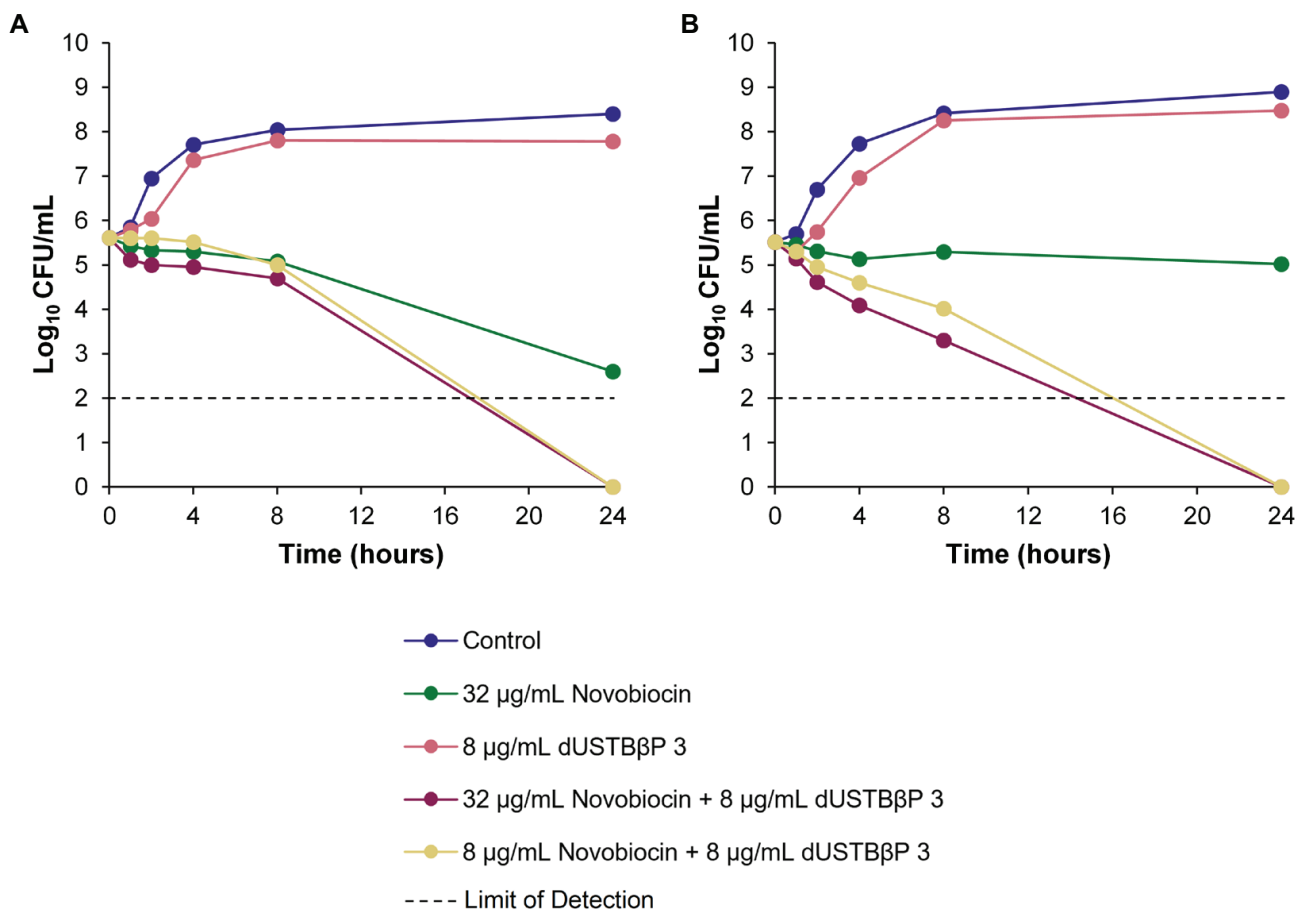
Time-kill kinetics of novobiocin alone and in combination with a fixed concentration of 8 μg/mL (6 μM) dUSTBβP 3 against **(A)** wild-type *A. baumannii* ATCC 17978 and **(B)** MDR *A. baumannii* 110193.

**Figure 4:**
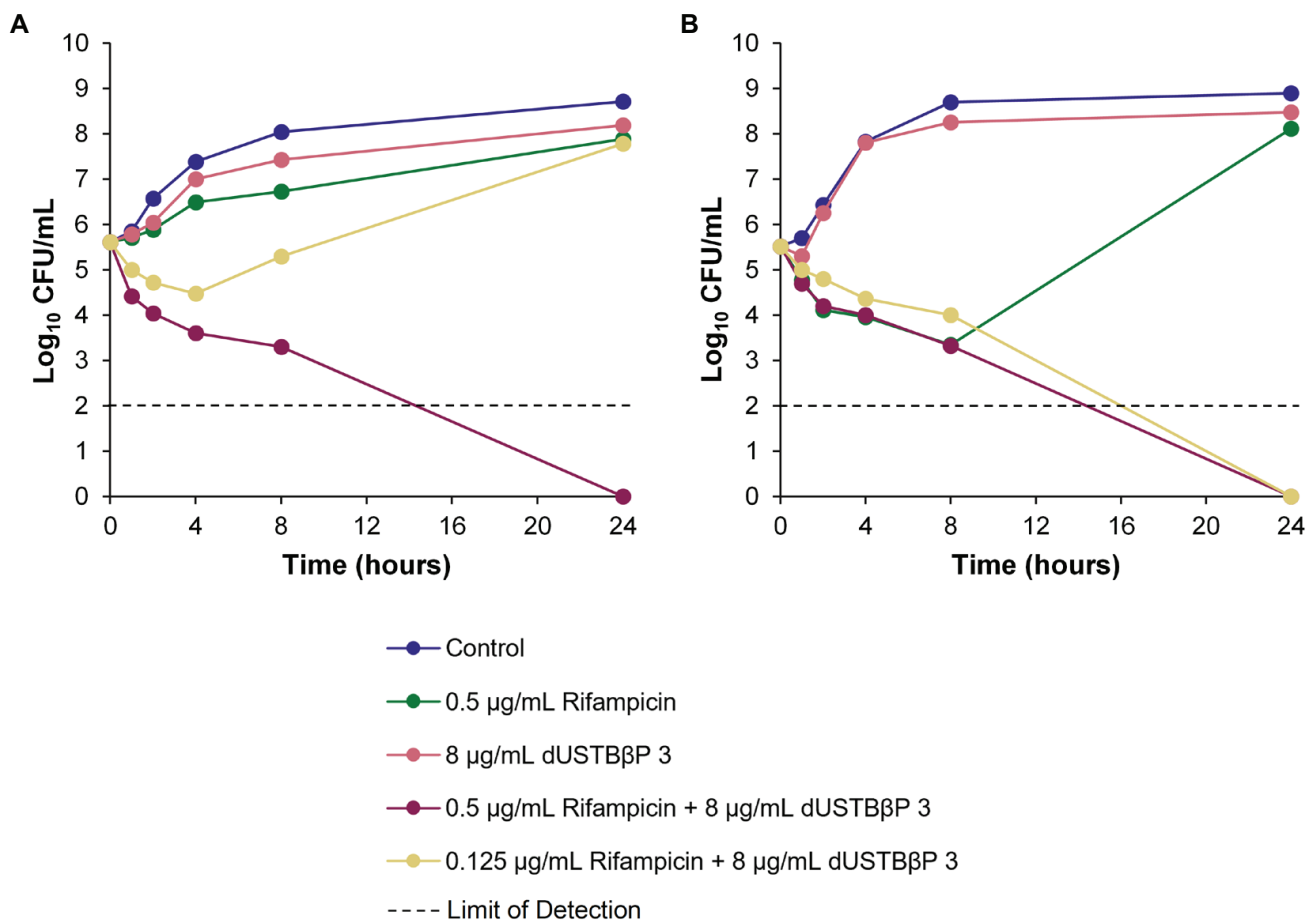
Time-kill kinetics of rifampicin alone and in combination with a fixed concentration of 8 μg/mL (6 μM) dUSTBβP 3 against **(A)** wild-type *A. baumannii* ATCC 17978 and **(B)** MDR *A. baumannii* 110193.

The ability of the combinations of dUSTBβP 3 and novobiocin (Figure 3) or rifampicin (Figure 4) to enhance the bacterial killing of wild-type *A. baumannii* ATCC 17978 and MDR *A. baumannii* 110193 was determined. Bactericidal and bacteriostatic activity is defined by *a* ≥ 3 log_10_ and *a* < 3 log_10_ decrease in CFU/mL from the original inoculum after 24 h, respectively (Clinical and Laboratory Standards Institute, 1999). Treatment of both strains with 8 μg/mL (6 μM) dUSTBβP 3 alone resulted in growth curves similar to the negative controls, indicating that compound 3 does not have intrinsic antibacterial activity. Bacteriostatic activity was exhibited by 32 μg/mL novobiocin or 0.5 μg/mL rifampicin in both wild-type and MDR *A. baumannii* strains. Combinations of 8 μg/mL (6 μM) compound 3 with either 8 or 32 μg/mL novobiocin lowered the bacterial load of both *A. baumannii* strains below the detection limit after 24 h. The addition of 8 μg/mL (6 μM) dUSTBβP 3 to either 0.125 or 0.5 μg/mL rifampicin sterilized *A. baumannii* 110193 after 24 h. While the combination of 8 μg/mL (6 μM) compound 3 and 0.125 μg/mL rifampicin was not able to suppress regrowth of *A. baumannii* ATCC 17978 after 4 h, increasing the rifampicin concentration to 0.5 μg/mL resulted in bactericidal effects after 24 h.

### dUSTBβP 3 Potentiated Multiple Classes of Antibiotics

**Figure 5:**
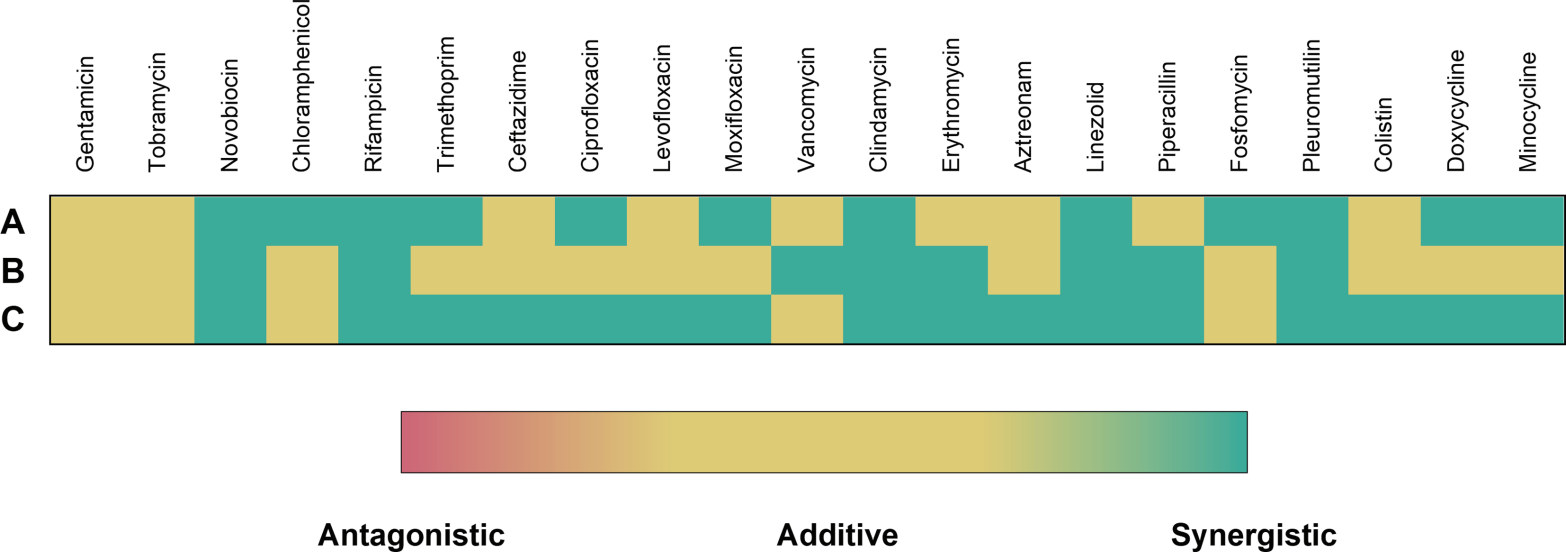
Interactions of dUSTBβP 3 at a fixed concentration of 8 μg/mL (6 μM) with different antibiotics against wild-type **(A)** *P. aeruginosa* PAO1, **(B)** *A. baumannii* ATCC 17978, and **(C)** *E. coli* ATCC 25922. FICI (FIC) ≤ 0.5 = Green; FICI > 0.5 but ≤4.0 = Yellow; and FICI > 4.0 = Red. See Supplementary Table 7 for MIC values of each combination.

In addition to novobiocin and rifampicin, synergy between dUSTBβP 3 and 19 other antibiotics were also screened against wild-type *P. aeruginosa*, *A. baumannii*, and *E. coli* (Figure 5). Synergy is defined by at least a 4-fold reduction in MIC of an antibiotic in combination with 8 μg/mL (6 μM) (>¼ × MIC of dUSTBβP 3) compound 3. dUSTBβP 3 potentiated multiple classes of antibiotics, including the aminocoumarins, ansamycins, antifolates, fluoroquinolones, lincosamides, macrolides, oxazolidinones, penicillins, pleuromutilins, and tetracyclines.

### dUSTBβP 3 Permeabilizes the OM

**Figure 6:**
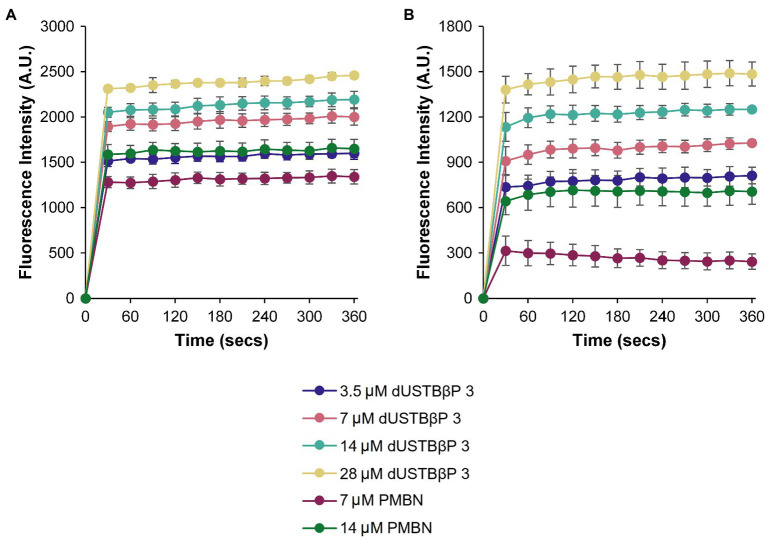
Measurement of OM permeabilization by dUSTBβP 3 through the accumulation of NPN in **(A)** *A. baumannii* ATCC 17978 and **(B)** *E. coli* ATCC 25922 cells. Control used was PMBN. Results were an average of triplicates (*n* = 3) ± SD.

To determine whether dUSTBβP 3 increases the intracellular concentration of novobiocin or rifampicin by permeabilizing the OM, the ability of the compound to increase the uptake of the nonpolar membrane-impermeable fluorescent probe NPN was measured in wild-type *A. baumannii* ATCC 17978 and *E. coli* ATCC 25922 (Figure 6). NPN uptake is normally prevented when the OM is intact (Idowu et al., 2019). Moreover, NPN fluoresces strongly and weakly in phospholipid and aqueous environments, respectively (Idowu et al., 2019). Since increasing concentrations of the OM permeabilizer PMBN or compound 3 resulted in increased fluorescence of NPN, this suggests that dUSTBβP 3 dose-dependently permeabilizes the OM.

### dUSTBβP 3 Displayed Enhanced Resistance to Proteases

**Figure 7:**
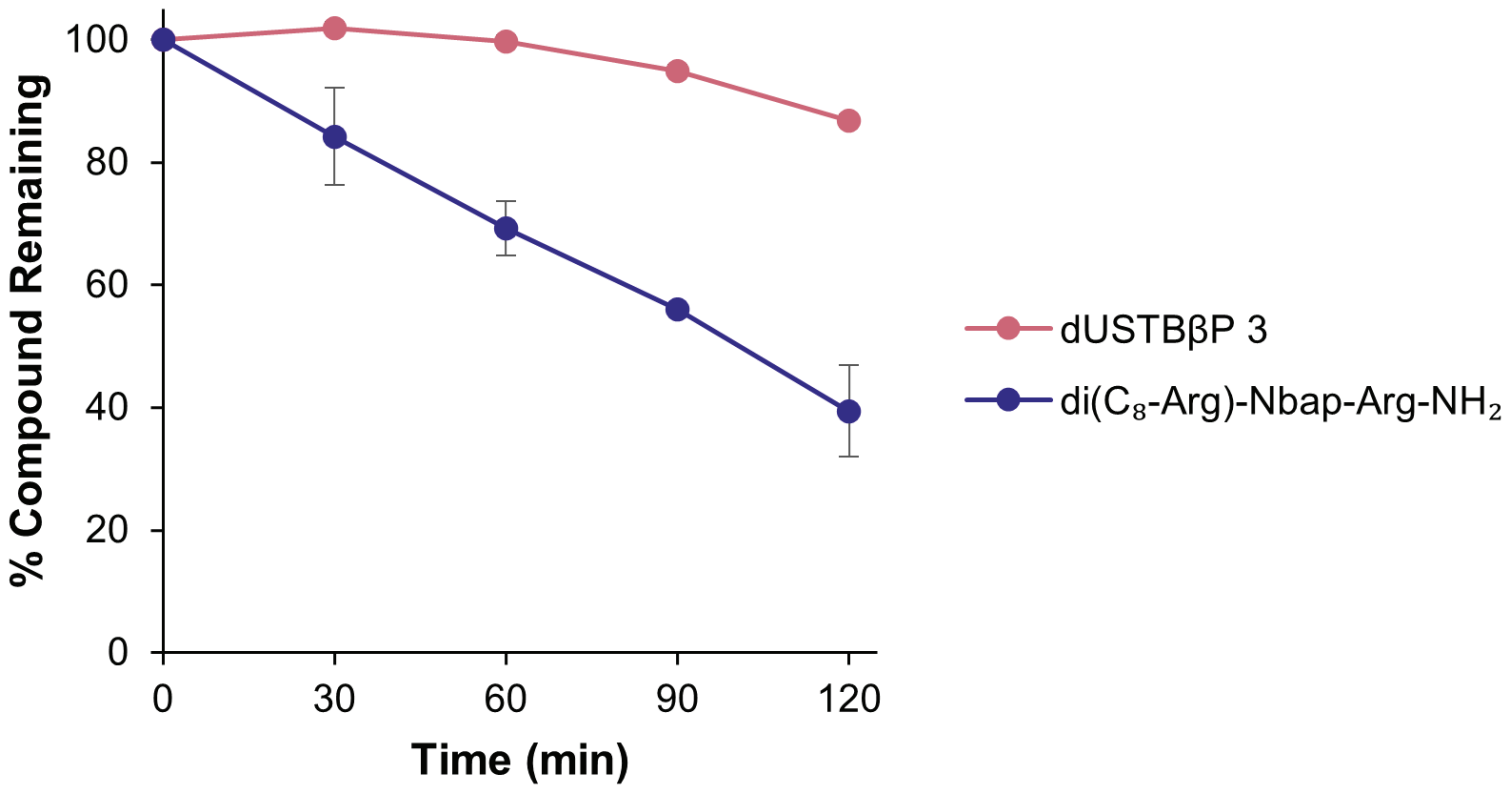
Stability profile of dUSTBβP 3 and di(C_8_-Arg)-*N*bap-Arg-NH_2_ in human plasma. Results were an average of duplicates (*n* = 2) ± SD.

Previously reported dUSTBP di(C_8_-Arg)-*N*bap-Arg-NH_2_ remained intact after incubation with trypsin for 2 h (Ramirez et al., 2020). Therefore, the ability of dUSTBβP 3 to resist tryptic degradation was also examined as an initial study of stability toward proteases. Compound 3 was incubated in the presence of trypsin for 2 h, and the resulting degradation mixture was subsequently assessed by electrospray ionization mass spectrometry molecular fragmentation analysis. To verify trypsin cleavage, dUSCL di-C_9_-KKKK-NH_2_ (Figure 1) was selected as a positive control. Solutions consisting of trypsin alone and peptide alone were chosen as negative controls. Complete degradation of the dUSCL occurred, as indicated by the loss of parent mass ions characteristic to untruncated di-C_9_-KKKK-NH_2_ (Supplementary Figure 1). However, resistance to trypsin was observed with the dUSTBβP, as parent mass ions corresponding to untruncated compound 3 were still present after 2 h (Supplementary Figure 2). Proteolytic stability was further evaluated by incubating dUSTBβP 3 in human plasma (Figure 7). Compound 3 (86.8 ± 3.7% remaining) was found to be stable for 2 h in plasma, unlike the α-amino acid-based derivative di(C_8_-Arg)-*N*bap-Arg-NH_2_ (39.5 ± 7.4% remaining).

### dUSTBβP 3 Is Noncytotoxic to Eukaryotic Cells

Toxicity of dUSTBβP 3 was evaluated against the eukaryotic HEK293 and HepG2 cell lines (Supplementary Figure 3). Testing of the anticancer drug doxorubicin was included as a positive control. Compound 3 at 125 μM and doxorubicin at 1 μM were noncytotoxic (86.4 ± 8.7% cell viability) and toxic (16.6 ± 2.3% cell viability) to HEK293 cells, respectively. Similarly, dUSTBβP 3 and doxorubicin exhibited limited activity (CC_50_ of 100.9 μM) and potent activity (CC_50_ of 0.032 μM) against HepG2 cells, respectively.

## Discussion

dUSTBβPs were designed based on our previously reported dUSTBPs (Figure 1). Basic amino acids were incorporated into the dUSTBP structure to achieve selective interaction with the anionic bacterial surface, two short hydrophobic fatty acyl tails for bacterial membrane destabilization, and the molecular scaffold *N*bap to improve proteolytic stability. Improved resistance to trypsin was observed with dUSTBP di(C_8_-Arg)-*N*bap-Arg-NH_2_ (Figure 1) in comparison to dUSCL di-C_9_-KKKK-NH_2_ (Figure 1; Ramirez et al., 2020). To further increase the resistance of dUSTBPs to proteases, β-amino acid-containing derivatives were produced. Particularly, β^3^-amino acids were used in which the sidechains are adjacent to the amine (Cabrele et al., 2014). Since β-amino acids contain an additional methylene in the backbone, interaction with protease active sites may be impeded, potentially resulting in decreased enzymatic degradation (Godballe et al., 2011).

All dUSTBβPs were produced by using SPPS on a Rink amide MBHA resin following an Fmoc protection strategy. Due to the nature of the MBHA resin, all synthesized compounds have an amidated C-terminus. The basic amino acids β^3^hOrn, β^3^hLys, or β^3^hArg were attached at three points on *N*bap. The N-terminus of both terminal amino acids was also acylated with C_8_ fatty acids, yielding three dUSTBβPs. Structural activity relationships studies revealed that C_8_ fatty acyl tails were relatively nonhemolytic and exhibited promising adjuvant potency by sensitizing Gram-negative bacteria to several antibiotics (Ramirez et al., 2020). However, fatty acyl tails four carbons long resulted to no potentiation possibly due to insufficient membrane interaction, and fatty acyl tails 12 carbons long exhibited hemolysis (Ramirez et al., 2020).

Selectivity of dUSTBβPs to bacterial cells rather than eukaryotic cells is a significant aspect to consider for clinical application. We previously reported di-C_8_ dUSTBPs to be nonhemolytic against porcine erythrocytes (Ramirez et al., 2020). Thus, we evaluated the propensity for hemolysis of the prepared compounds on human erythrocytes (Figure 2). Red blood cells were subjected to dUSTBβPs at concentrations ranging from 1.5625 to 200 μM. Even at the highest concentration tested, the compounds were found to be nonhemolytic (<5% hemolysis).

Susceptibility of wild-type *P. aeruginosa*, *A. baumannii*, and *E. coli* to dUSTBβPs was also assessed (Table 1). Similar to the di-C_8_ α-amino acid-based derivatives, the compounds did not display intrinsic antibacterial activity (Ramirez et al., 2020). While previous studies have shown that C_8_ fatty acyl tails were not sufficient to confer inherent activity, they displayed potent synergy with novobiocin and rifampicin against wild-type and MDR clinical isolates of Gram-negative bacteria (Ramirez et al., 2020). Therefore, the adjuvant properties of dUSTBβPs were also investigated.

The di-C_8_ dUSTBPs acted as adjuvants that consistently enhanced the antibacterial activity of novobiocin and rifampicin against wild-type Gram-negative bacteria (Ramirez et al., 2020). Hence, the ability of dUSTBβPs to also potentiate these two antibiotics against *P. aeruginosa* PAO1 (Table 2), *A. baumannii* ATCC 17978 (Table 3), and *E. coli* ATCC 25922 (Table 4) was studied. In general, the synthesized compounds displayed similar novobiocin and rifampicin potentiation in comparison with their α-amino acid-based counterparts. Out of the three compounds, dUSTBβP 3 (with β^3^hArg residues) proved most promising. For instance, compound 3 reduced the MIC of novobiocin and rifampicin 4- to 32-fold better than the β^3^hOrn and β^3^hLys derivatives. This matches previous structural activity relationship studies that have shown that guanidino functions may confer preferential membrane activity compared to primary amines (Nakase et al., 2012; Andreev et al., 2014; Ramirez et al., 2020). As such, the adjuvant activity of compound 3 with novobiocin and rifampicin was further explored against MDR Gram-negative bacteria.

Potentiation of novobiocin (Table 5) or rifampicin (Table 6) by dUSTBβP 3 was examined against MDR clinical isolates of *P. aeruginosa*, *A. baumannii*, *E. coli*, *Klebsiella pneumoniae*, and *Enterobacter cloacae*. Relative to the α-amino acid-based derivative di(C_8_-Arg)-*N*bap-Arg-NH_2_, a slight reduction in antibiotic potentiation was observed with compound 3 against *P. aeruginosa* while an increase in rifampicin potentiation was observed against *A. baumannii* and *E. coli*. For instance, 8 μg/mL (6 μM) compound 3 potentiated rifampicin 8-fold less in PA264-104354 and PA91433. Moreover, 8 μg/mL (6 μM) dUSTBβP 3 potentiated rifampicin between 2- and 8-fold more than di(C_8_-Arg)-*N*bap-Arg-NH_2_ against *A. baumannii* and *E. coli*, except against *E. coli* strain 107115.

The ability of dUSTBβP 3 to reduce absolute MICs of novobiocin and rifampicin below their clinical breakpoints was evaluated. Currently, both the CLSI and the European Committee on Antimicrobial Susceptibility Testing have no breakpoint values listed for these two antibiotics against *P. aeruginosa*, *A. baumannii*, and *Enterobacteriaceae*. Thus, established breakpoints for other organisms were cautiously selected as reference. For rifampicin, a CLSI susceptibility breakpoint of ≤1 μg/mL for *Staphylococcus* spp. was used (Clinical and Laboratory Standards Institute, 2019). For novobiocin, an interpretative susceptibility breakpoint of 4 μg/mL based on bovine mastitis pathogens was used (Thornsberry et al., 1997). It has also been previously reported that novobiocin and colistin combination therapy which potentiates novobiocin to below the steady-state serum concentration of 5 μg/mL may have clinical potential (MacNair et al., 2018). In combination with 8 μg/mL (6 μM) compound 3, the MIC of novobiocin was reduced below the susceptibility breakpoint in all *A. baumannii*, *E. coli*, *K. pneumoniae*, and *E. cloacae* strains (Table 5). Moreover, MICs of rifampicin below the clinical breakpoint were achieved in two of the five *P. aeruginosa* strains, all *A. baumannii* strains tested, and eight of the nine *Enterobacteriaceae* strains (Table 6). Indeed, these results indicate that dUSTBβP 3 is a potent adjuvant for novobiocin and rifampicin against MDR Gram-negative bacteria. The drastic potentiation of rifampicin in *A. baumannii* is of particular note. A concentration of 8 μg/mL SPR741 has previously been shown to reduce the MIC of rifampicin against *A. baumannii* to between 0.002 and 0.03 μg/mL against 25 clinical isolates (Corbett et al., 2017). All observed MIC values for rifampicin in combination with compound 3 fall within the same range, indicating that dUSTBβP 3 displays comparable potentiation with SPR741 (Corbett et al., 2017). To study the bacteriostatic or bactericidal activity of the combinations of compound 3 and the two antibiotics, time-kill kinetics against *A. baumannii* were conducted.

The combinations of dUSTBP di(C_8_-Arg)-*N*bap-Arg-NH_2_ and novobiocin or rifampicin were previously described to enhance the bacterial killing of wild-type *A. baumannii* ATCC 17978 and MDR *A. baumannii* 110193 (Ramirez et al., 2020). To determine whether this effect is conserved with dUSTBβP 3, time-kill assays were performed against the same strains (Figures 3, 4). Interestingly, the combination of 0.5 μg/mL rifampicin with 8 μg/mL (6 μM) di(C_8_-Arg)-*N*bap-Arg-NH_2_ resulted in complete eradication of the wild-type strain in just 8 h (Ramirez et al., 2020). Whereas the combination of 0.5 μg/mL rifampicin with 8 μg/mL (6 μM) compound 3 only reached the same effect past 8 h. This suggests that the β-amino acid-containing derivative possibly exhibits slower killing kinetics than its α-amino acid-based counterpart. Overall, our data indicate that enhanced bacterial killing of both *A. baumannii* strains can be achieved with combination therapy consisting of dUSTBβP 3 and novobiocin or rifampicin.

Potential for synergy between dUSTBβP 3 and other clinically relevant antibiotics was also examined in wild-type Gram-negative bacteria (Figure 5). Tested antibiotics include, but are not limited to, those that have minimal activity against Gram-negative bacteria but are potent against Gram-positive bacteria. Thus, dUSTBβP 3 synergized with antibiotics considered OM-impermeable (novobiocin, rifampicin, vancomycin, clindamycin, erythromycin, and linezolid) and efflux-susceptible (chloramphenicol, trimethoprim, ceftazidime, ciprofloxacin, levofloxacin, moxifloxacin, clindamycin, aztreonam, linezolid, piperacillin, fosfomycin, pleuromutilin, doxycycline, and minocycline). Of the 21 tested antibiotics, compound 3 potentiated 12, eight, and 16 in PAO1, *A. baumannii* ATCC 17978, and *E. coli* ATCC 25922, respectively. Besides novobiocin and rifampicin, clindamycin and pleuromutilin were also consistently potentiated against all tested wild-type strains. These initial results suggest that dUSTBβPs increase the uptake of antibiotics by permeabilizing the OM, as well as by potentially suppressing efflux. Future investigation will explore the precise mode of action to confirm these results.

To elucidate the mechanism of antibiotic potentiation, the ability of dUSTBβP 3 to permeabilize the OM was initially studied in *A. baumannii* ATCC 17978 and *E. coli* ATCC 25922 (Figure 6). The two wild-type strains were selected since the combinations of compound 3 with novobiocin or rifampicin were most active against these organisms. NPN assay was performed using compound 3 at concentrations ranging from 3.5 to 28 μM. Testing of the known OM permeabilizer PMBN was included as a positive control. The data confirm the synergy displayed in the checkerboard studies, and that compound 3 permeabilizes the OM of both strains in a concentration-dependent manner. Moreover, at equimolar concentrations (7 and 14 μM), dUSTBβP 3 caused higher NPN fluorescence than PMBN.

The systemic usage of peptide therapeutics has been limited due to the poor bioavailability and proteolytic susceptibility associated with these molecules (Latham, 1999). To overcome these challenges, peptidomimetic strategies such as backbone modifications and the integration of unnatural amino acids can be employed (Avan et al., 2014). We have formerly shown that the incorporation of the *N*-substituted glycine *N*bap to the dUSTBP design resulted in increased stability toward trypsin (Ramirez et al., 2020). β^3^-amino acids were introduced herein into the lead dUSTBP structure to improve resistance to nonspecific proteolysis. To assess the proteolytic stability of dUSTBβP 3, the ability of the compound to resist degradation by trypsin and in human plasma was assessed (Figure 7; Supplementary Figure 2). After 2 h incubation in both conditions, it was found that compound 3 was stable. In addition, compound 3 has an extrapolated *in vitro* plasma half-life (10.43 ± 3.70 h) comparable with the *in vivo* elimination half-lives of three of the seven AMPs approved by the Food and Drug Administration: colistin (5 h), daptomycin (8–9 h), and the lypoglycopeptide telavancin (8 h; Chen and Lu, 2020). These results indicate that employing multiple peptidomimetic strategies into one molecule can result in enhanced resistance to proteases.

The effect of dUSTBβPs on eukaryotic cells was initially screened against human erythrocytes (Figure 2). dUSTBβP 3, which was found to be nonhemolytic, showed the greatest adjuvant potency out of the three derivatives. Therefore, the probability of dUSTBβP 3 to induce cytotoxicity to HEK293 and HepG2 human cells was further examined (Supplementary Figure 3). Cell viability assay was performed at concentrations ranging from 1 to 125 μM. At the highest concentration tested, dUSTBβP 3 was noncytotoxic to HEK293 cells (86.4% ± 8.7% cell viability). In contrast, dUSTBβP 3 has a CC_50_ (100.9 μM) against HepG2 cells 17-fold higher than the effective adjuvant concentration (6 μM) used in the synergistic studies.

To improve resistance to nonspecific proteolytic degradation, three β-amino acid-containing derivatives of dUSTBPs were prepared. In comparison to the α-amino acid-based counterpart, novobiocin and rifampicin potentiation by compound 3 were conserved against wild-type and MDR clinical isolates of *P. aeruginosa*, *A. baumannii*, and *Enterobacteriaceae*. The nonhemolytic dUSTBβP 3, consisting of β^3^hArg residues, lowered the MICs of novobiocin and rifampicin below their interpretative susceptibility breakpoints. Furthermore, compound 3 showed excellent *in vitro* plasma stability with an extrapolated half-life of 10.43 ± 3.70 h.

## Data Availability Statement

The original contributions presented in the study are included in the article/**Supplementary Material**; further inquiries can be directed to the corresponding author.

## Author Contributions

DR and FS conceived the study. DR prepared the compounds and performed the antimicrobial susceptibility, checkerboard, time-kill, and tryptic digest assays. DR and LB characterized the compounds. YL and DR performed the NPN assays. GA performed the cell viability assays. FS and AK supervised the microbiological and biochemical assays. DR, LB, RD, and FS wrote the manuscript. All authors contributed to the article and approved the submitted version.

## Funding

This work was supported by the Canadian Institutes of Health Research (CIHR) in the form of a pilot project (162159) and the Natural Sciences and Engineering Research Council of Canada (NSERC) in the form of a discovery grant (2018-06047).

## Conflict of Interest

The authors declare that the research was conducted in the absence of any commercial or financial relationships that could be construed as a potential conflict of interest.

## Publisher’s Note

All claims expressed in this article are solely those of the authors and do not necessarily represent those of their affiliated organizations, or those of the publisher, the editors and the reviewers. Any product that may be evaluated in this article, or claim that may be made by its manufacturer, is not guaranteed or endorsed by the publisher.
