# Supplementary material for: Dioctanoyl Ultrashort Tetrabasic β-Peptides Sensitize Multidrug-Resistant Gram-Negative Bacteria to Novobiocin and Rifampicin
Source: Front Microbiol. 2021 Dec 23;12:803309. doi: 10.3389/fmicb.2021.803309 (PMC8733726; doi:10.3389/fmicb.2021.803309)
Supplement: Supplementary file 1 [file Data_Sheet_1.pdf]

Supplementary Material

Table of Contents

1. Chemical characterization of dUSTBβPs 2

2. Molecular weight of dUSTBβPs in salt form 5

3. Hemolytic activity of dUSTBβPs 5

4. Synergistic studies of dUSTBβP **3** with novobiocin or rifampicin against MDR Gram-negative bacteria 6

5. Synergistic studies of dUSTBβP **3** with other antibiotics against wild-type Gram-negative bacteria 9

6. Tryptic digest of dUSCL di-C_9-KKKK-NH_2 and dUSTBβP **3** 10

7. Cytotoxicity of dUSTBβP **3** 12

8. NMR spectra of dUSTBβPs 12

9. HPLC chromatograms of dUSTBβPs...20

10. Resistance phenotype of MDR clinical isolates 23

1 Chemical characterization of dUSTBβPs

Dioctanoyl-diβ^{3}hOrn-bis(3-aminopropyl)glycine-β^{3}hOrn-NH_2 (1).

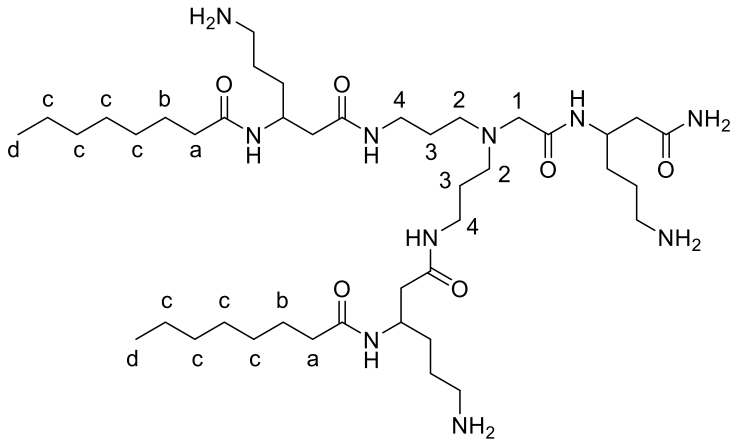

^{1}H NMR (500 MHz, Methanol-d_4) δ 4.35 – 4.28 (m, 1H, Orn_1-β), 4.28 – 4.19 (m, 2H, Orn_2-β + Orn_3-β), 4.05 – 3.87 (m, 2H, Linker-1), 3.38 – 3.30 (m, 0.5H, Linker-2 + Linker-4), 3.29 – 3.08 (m, 7.5H, Linker-2 + Linker-4), 3.02 – 2.88 (m, 6H, Orn_1-ε + Orn_2-ε + Orn_3-ε), 2.53 – 2.29 (m, 6H, Orn_1-α + Orn_2-α + Orn_3-α), 2.24 – 2.10 (m, 4H, Aliphatic-a), 1.98 – 1.79 (m, 4H, Linker-3), 1.76 – 1.48 (m, 16H, Aliphatic-b + Orn_1-γ + Orn_2-γ + Orn_3-γ + Orn_1-δ + Orn_2-δ + Orn_3-δ), 1.37 – 1.21 (m, 16H, Aliphatic-c), 1.00 – 0.74 (m, 6H, Aliphatic-d).

^{13}C NMR (126 MHz, Methanol-d_4) δ 174.68, 174.09, 172.59, 164.45, 53.33 (Linker-1), 53.14 (Linker-2 + Linker-4), 46.67 (Orn_1-β), 46.05 (Orn_2-β + Orn_3-β), 41.13 (Orn_2-α + Orn_3-α), 39.87 (Orn_1-α), 39.01 (Orn_1-ε + Orn_2-ε + Orn_3-ε), 38.94 (Orn_1-ε + Orn_2-ε + Orn_3-ε), 35.91 (Aliphatic-a), 35.65 (Linker-2 + Linker-4), 31.44 (Aliphatic-b + Orn_1-γ + Orn_2-γ + Orn_3-γ + Orn_1-δ + Orn_2-δ + Orn_3-δ), 31.16 (Aliphatic-b + Orn_1-γ + Orn_2-γ + Orn_3-γ + Orn_1-δ + Orn_2-δ + Orn_3-δ), 30.98 (Aliphatic-b + Orn_1-γ + Orn_2-γ + Orn_3-γ + Orn_1-δ + Orn_2-δ + Orn_3-δ), 28.95 (Aliphatic-c), 28.69 (Aliphatic-c), 25.73 (Aliphatic-b + Orn_1-γ + Orn_2-γ + Orn_3-γ + Orn_1-δ + Orn_2-δ + Orn_3-δ), 24.15 (Linker-3), 23.80 (Aliphatic-b + Orn_1-γ + Orn_2-γ + Orn_3-γ + Orn_1-δ + Orn_2-δ + Orn_3-δ), 23.73 (Aliphatic-c), 22.24 (Aliphatic-c), 12.99 (Aliphatic-d).

MS (+TOF) m/z: calculated for $C_{42}H_{84}N_{10}O_6$ $[M+H]^+$: 825.665, found: 825.754; $[M+Na]^+$: 847.647, found: 847.776.

Dioctanoyl-diβ^{3}hLys-bis(3-aminopropyl)glycine-β^{3}hLys-NH$_2$ (2).

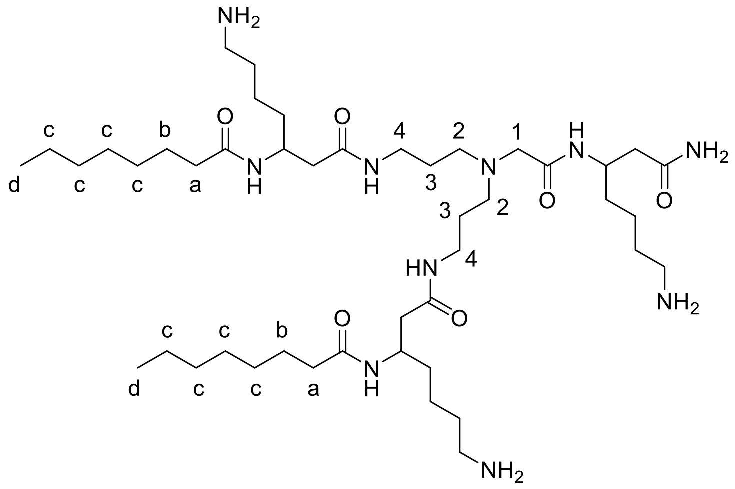

^{1}H NMR (500 MHz, Methanol-d_4) δ 4.32 – 4.26 (m, 1H, Lys$_1$-β), 4.26 – 4.19 (m, 2H, Lys$_2$-β + Lys$_3$-β), 4.06 – 3.99 (m, 1H, Linker-1), 3.94 – 3.85 (m, 1H, Linker-1), 3.33 – 3.30 (m, 0.5H, Linker-2 + Linker-4), 3.29 – 3.15 (m, 7.5H, Linker-2 + Linker-4), 2.96 – 2.86 (m, 6H, Lys$_1$-ω + Lys$_2$-ω + Lys$_3$-ω), 2.49 – 2.30 (m, 6H, Lys$_1$-α + Lys$_2$-α + Lys$_3$-α), 2.17 (m, 4H, Aliphatic-a), 1.97 – 1.82 (m, 4H, Linker-3), 1.73 – 1.63 (m, 6H, Lys$_1$-ε + Lys$_2$-ε + Lys$_3$-ε), 1.62 – 1.48 (m, 10H, Aliphatic-b, Lys$_1$-γ + Lys$_2$-γ + Lys$_3$-γ), 1.47 – 1.35 (m, 6H, Lys$_1$-δ + Lys$_2$-δ + Lys$_3$-δ), 1.34 – 1.23 (m, 16H, Aliphatic-c), 0.94 – 0.82 (m, 6H, Aliphatic-d).

^{13}C NMR (126 MHz, Methanol-d_4) δ 174.54, 174.26, 172.75, 164.15, 53.39 (Linker-2 + Linker-4), 53.25 (Linker-1), 47.07 (Lys$_1$-β), 46.35 (Lys$_2$-β + Lys$_3$-β), 41.24 (Lys$_2$-α + Lys$_3$-α), 39.92 (Lys$_1$-α), 39.14 (Lys$_1$-ω + Lys$_2$-ω + Lys$_3$-ω), 39.09 (Lys$_1$-ω + Lys$_2$-ω + Lys$_3$-ω), 35.90 (Aliphatic-a), 35.60 (Linker-2 + Linker-4), 33.70 (Aliphatic-b + Lys$_1$-γ + Lys$_2$-γ + Lys$_3$-γ), 33.48 (Aliphatic-b + Lys$_1$-γ + Lys$_2$-γ + Lys$_3$-γ), 31.48 (Aliphatic-c), 28.70 (Aliphatic-c), 26.78 (Aliphatic-c), 26.73 (Lys$_1$-ε + Lys$_2$-ε + Lys$_3$-ε), 26.67 (Lys$_1$-ε + Lys$_2$-ε + Lys$_3$-ε), 25.76 (Aliphatic-b + Lys$_1$-γ + Lys$_2$-γ + Lys$_3$-γ), 24.13 (Linker-3), 22.53 (Lys$_1$-δ + Lys$_2$-δ + Lys$_3$-δ), 22.45 (Lys$_1$-δ + Lys$_2$-δ + Lys$_3$-δ), 22.24 (Aliphatic-c), 12.99 (Aliphatic-d).

MS (+TOF) m/z: calculated for $C_{45}H_{90}N_{10}O_6$ $[M+H]^+$: 867.712, found: 867.734; $[M+Na]^+$: 889.694, found: 889.713.

Dioctanoyl-diβ^{3}hArg-bis(3-aminopropyl)glycine-β^{3}hArg-NH$_2$ (3).

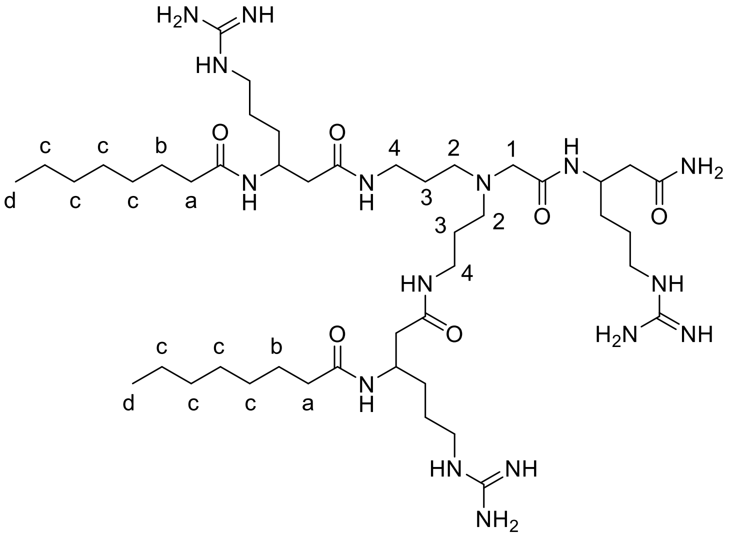

^{1}H NMR (500 MHz, Methanol-d_4) δ 4.36 – 4.28 (m, 1H, Arg$_1$-β), 4.21 – 4.15 (m, 2H, Arg$_2$-β + Arg$_3$-β), 4.11 – 4.00 (m, 2H, Linker-1), 3.39 – 3.31 (m, 2H, Linker-2 + Linker-4 + Arg$_1$-ε + Arg$_2$-ε + Arg$_3$-ε), 3.27 – 3.16 (m, 12H, Linker-2 + Linker-4 + Arg$_1$-ε + Arg$_2$-ε + Arg$_3$-ε), 2.30 – 2.22 (m, 4H, Aliphatic-a), 1.97 – 1.89 (m, 4H, Linker-3), 1.87 – 1.67 (m, 6H, Arg$_1$-α + Arg$_2$-α + Arg$_3$-α), 1.64 – 1.57 (m, 10H, Aliphatic-b + Arg$_1$-δ + Arg$_2$-δ + Arg$_3$-δ), 1.51 – 1.37 (m, 6H, Arg$_1$-γ + Arg$_2$-γ + Arg$_3$-γ), 1.33 – 1.25 (m, 16H, Aliphatic-c), 0.91 – 0.87 (m, 6H, Aliphatic-d).

^{13}C NMR (126 MHz, Methanol-d_4) δ 175.24, 174.85, 174.01, 164.91, 157.28, 157.23, 53.84 (Arg$_2$-β + Arg$_3$-β), 53.60 (Arg$_1$-β), 53.54 (Linker-1), 53.13 (Linker-2 + Linker-4), 40.73 (Arg$_2$-ε + Arg$_3$-ε), 40.67 (Arg$_1$-ε), 35.56 (Linker-2 + Linker-4), 35.39 (Aliphatic-a), 31.48 (Aliphatic-c), 31.14 (Arg$_1$-α), 30.91 (Arg$_2$-α + Arg$_3$-α), 28.95 (Aliphatic-c), 28.73 (Aliphatic-c), 27.94 (Aliphatic-b + Arg$_1$-δ + Arg$_2$-δ + Arg$_3$-δ), 25.50 (Arg$_1$-δ + Arg$_2$-δ + Arg$_3$-δ), 23.90 (Linker-3), 22.74 (Arg$_2$-γ + Arg$_3$-γ), 22.58 (Aliphatic-b + Arg$_1$-δ + Arg$_2$-δ + Arg$_3$-δ), 22.54 (Arg$_1$-γ), 22.24 (Aliphatic-c), 12.99 (Aliphatic-d).

MS (+TOF) m/z: calculated for $C_{45}H_{90}N_{16}O_6$ $[M+H]^+$: 951.730, found: 951.815; $[M+Na]^+$: 973.713, found: 973.780.

2 Molecular weight of dUSTBβPs in salt form

Supplementary Table 1. Molecular weight of dUSTBβPs in TFA salt form.

dUSTBβP (x 4TFA)	Molecular weight (g/mol)
**1**	1281.2912
**2**	1323.3722
**3**	1407.4142

3 Hemolytic activity of dUSTBβPs

Supplementary Table 2. Concentration-dependent hemolytic activity of dUSTBβPs against red blood cells.

dUSTBβP	% Hemolysis (relative to control[a]) elicited at respective concentration							
	200 μM	100 μM	50 μM	25 μM	12.5 μM	6.25 μM	3.125 μM	1.5625 μM
**1**	2.26 ± 0.19	3.35 ± 0.17	3.17 ± 0.06	3.12 ± 0.05	3.16 ± 0.04	3.13 ± 0.00	3.12 ± 0.09	3.11 ± 0.07
**2**	3.21 ± 0.13	3.26 ± 0.10	3.24 ± 0.15	3.29 ± 0.14	3.22 ± 0.20	3.23 ± 0.11	3.28 ± 0.21	3.33 ± 0.12
**3**	4.59 ± 2.42	4.17 ± 1.29	3.43 ± 0.49	3.35 ± 0.09	3.28 ± 0.12	3.18 ± 0.05	3.32 ± 0.16	3.23 ± 0.08

[a]Control used was 1% Triton X-100

Supplementary Table 3. Hemolytic activity of controls against red blood cells.

Controls	% Hemolysis
0.1% Triton X-100	8.01 ± 0.18
1% Triton X-100	99.17 ± 3.06
Vehicle[a]	3.05 ± 0.04
Untreated	3.39 ± 0.07

[a]Saline + 10% DMSO (final DMSO concentration in assay = 1%)

4 Synergistic studies of dUSTBβP 3 with novobiocin or rifampicin against MDR Gram-negative bacteria

Supplementary Table 4. Synergy evaluation of combinations consisting of dUSTBβP **3** and novobiocin or rifampicin against MDR clinical isolates of *P. aeruginosa*.

**Organism**	**Antibiotic**	$MIC_{dUSTB\beta P}$ [MIC_{combo}] (μg/mL)	$MIC_{antibiotic}$ [MIC_{combo}] (μg/mL)	FICI	Interpretation	Absolute $MIC^{a}_{antibiotic}$ (μg/mL)	Potentiation[b]
P. aeruginosa PA259-96196	**Novobiocin**	>128 [8]	1024 [32]	0.031<x<0.094	Synergy	32	32-fold
	**Rifampicin**	>128 [8]	16 [0.5]	0.031<x<0.094	Synergy	0.5	32-fold
P. aeruginosa PA262-101856	**Novobiocin**	>128 [16]	1024 [16]	0.016<x<0.141	Synergy	1024	1-fold
	**Rifampicin**	>128 [8]	512 [64]	0.125<x<0.188	Synergy	64	8-fold
P. aeruginosa PA264-104354	**Novobiocin**	>128 [16]	1024 [16]	0.016<x<0.141	Synergy	512	2-fold
	**Rifampicin**	>128 [8]	16 [0.5]	0.031<x<0.094	Synergy	0.5	32-fold
P. aeruginosa PA91433	**Novobiocin**	>128 [16]	1024 [16]	0.016<x<0.141	Synergy	1024	1-fold
	**Rifampicin**	>128 [16]	16 [2]	0.125<x<0.25	Synergy	8	2-fold
P. aeruginosa PA114228	**Novobiocin**	>128 [16]	1024 [128]	0.125<x<0.25	Synergy	256	4-fold
	**Rifampicin**	>128 [16]	16 [4]	0.25<x<0.375	Synergy	8	2-fold

[a]MIC of antibiotic in the presence of 8 μg/mL (6 μM) dUSTBβP **3**

[b]Degree of antibiotic potentiation in the presence 8 μg/mL (6 μM) dUSTBβP **3**

Supplementary Table 5. Synergy evaluation of combinations consisting of dUSTBβP **3** and novobiocin or rifampicin against MDR clinical isolates of *A. baumannii*.

**Organism**	**Antibiotic**	$MIC_{dUSTB\beta P}$ [MIC_{combo}] (μg/mL)	$MIC_{antibiotic}$ [MIC_{combo}] (μg/mL)	FICI	Interpretation	Absolute $MIC^{a}_{antibiotic}$ (μg/mL)	Potentiation[b]
A. baumannii AB027	**Novobiocin**	>128 [4]	8 [0.063]	0.008<x<0.039	Synergy	0.063	128-fold
	**Rifampicin**	>128 [8]	1 [0.008]	0.008<x<0.070	Synergy	0.008	128-fold
A. baumannii AB031	**Novobiocin**	>128 [8]	4 [0.031]	0.008>x>0.070	Synergy	0.031	128-fold
	**Rifampicin**	>128 [8]	1 [0.002]	0.002<x<0.064	Synergy	0.002	512-fold
A. baumannii LAC-4	**Novobiocin**	16 [4]	>0.5[0.016]	x<0.281	Synergy	0.002	>256-fold
	**Rifampicin**	16 [4]	0.5 [0.016]	0.281	Synergy	0.004	128-fold
A. baumannii 92247	**Novobiocin**	128 [4]	4 [0.063]	0.047	Synergy	0.063	64-fold
	**Rifampicin**	128 [8]	2 [0.016]	0.070	Synergy	0.016	128-fold
A. baumannii 110193	**Novobiocin**	>128 [8]	128 [0.125]	0.001<x<0.063	Synergy	0.125	1024-fold
	**Rifampicin**	>128 [8]	1 [0.008]	0.008<x<0.070	Synergy	0.008	128-fold

[a]MIC of antibiotic in the presence of 8 μg/mL (6 μM) dUSTBβP **3**

[b]Degree of antibiotic potentiation in the presence 8 μg/mL (6 μM) dUSTBβP **3**

Supplementary Table 6. Synergy evaluation of combinations consisting of dUSTBβP **3** and novobiocin or rifampicin against MDR clinical isolates of *Enterobacteriaceae*.

**Organism**	**Antibiotic**	$MIC_{dUSTB\beta P}$ [MIC_{combo}] (μg/mL)	$MIC_{antibiotic}$ [MIC_{combo}] (μg/mL)	FICI	Interpretation	Absolute $MIC^{a}_{antibiotic}$ (μg/mL)	Potentiation[b]
E. coli 94393	**Novobiocin**	64 [8]	64 [0.125]	0.127	Synergy	0.125	512-fold
	**Rifampicin**	64 [8]	8 [0.016]	0.127	Synergy	0.016	512-fold
E. coli 94474	**Novobiocin**	128 [8]	256 [0.5]	0.064	Synergy	0.5	512-fold
	**Rifampicin**	128 [8]	8 [0.008]	0.063	Synergy	0.008	1024-fold
E. coli 107115	**Novobiocin**	64 [2]	128 [1]	0.039	Synergy	0.5	256-fold
	**Rifampicin**	64 [8]	32 [0.004]	0.125	Synergy	0.004	8192-fold
K. pneumoniae 113250	**Novobiocin**	>128 [8]	128 [2]	0.016<x<0.078	Synergy	2	64-fold
	**Rifampicin**	>128 [4]	32 [1]	0.031<x<0.063	Synergy	1	32-fold
K. pneumoniae 113254	**Novobiocin**	>128 [8]	256 [4]	0.016<x<0.078	Synergy	4	64-fold
	**Rifampicin**	>128 [4]	8 [0.5]	0.063<x<0.094	Synergy	0.5	16-fold
K. pneumoniae 116381	**Novobiocin**	>128 [8]	256 [1]	0.004<x<0.066	Synergy	1	256-fold
	**Rifampicin**	>128 [4]	>256 [32]	x<0.156	Synergy	32	>8-fold
E. cloacae 117029	**Novobiocin**	>128 [8]	512 [0.25]	0.0005<x<0.063	Synergy	0.25	2048-fold
	**Rifampicin**	>128 [8]	8 [0.004]	0.0005<x<0.063	Synergy	0.004	2048-fold
E. cloacae 118564	**Novobiocin**	>128 [8]	256 [0.5]	0.002<x<0.064	Synergy	0.5	512-fold
	**Rifampicin**	>128 [8]	8 [0.125]	0.016<x<0.078	Synergy	0.125	64-fold
E. cloacae 121187	**Novobiocin**	>128 [4]	16 [2]	0.125<x<0.156	Synergy	2	8-fold
	**Rifampicin**	>128 [8]	2 [0.25]	0.125<x<0.188	Synergy	0.25	8-fold

[a]MIC of antibiotic in the presence of 8 μg/mL (6 μM) dUSTBβP **3**

[b]Degree of antibiotic potentiation in the presence 8 μg/mL (6 μM) dUSTBβP **3**

5 Synergistic studies of dUSTBβP 3 with other antibiotics against wild-type Gram-negative bacteria

Supplementary Table 7. Combination studies of dUSTBβPs **3** (at 8 µg/mL) and twenty-one antibiotics against *Pseudomonas aeruginosa* PAO1, *Acinetobacter baumannii* ATCC 17978 and *Escherichia coli* ATCC 25922. MIC reduction of ≥ 4-fold = synergistic, MIC increase of > 4-fold = antagonistic, no change or < 4-fold change in MIC = additive. Synergistic combinations are highlighted in green.

	$MIC_{antibiotic}$ [MIC_{combo}]		
	**WT *P. aeruginosa***	**WT *A. baumannii***	**WT *E. coli***
Gentamicin	2 [2]	>1 [>1]	2 [1]
Tobramycin	1 [1]	>1 [>1]	2 [1]
Novobiocin	1024 [32]	16 [0.25]	64 [0.125]
Chloramphenicol	32 [2]	128 [64]	2 [1]
Rifampicin	16 [0.25]	2 [0.008]	4 [0.008]
Trimethoprim	128 [32]	>128 [>128]	512 [64]
Ceftazidime	2 [2]	16 [8]	0.25 [0.063]
Ciprofloxacin	0.125 [0.031]	1 [1]	0.008 [0.002]
Levofloxacin	0.5 [0.25]	0.25 [0.25]	0.008 [0.002]
Moxifloxacin	2 [0.5]	>0.125 [>0.125]	0.016 [0.001]
Vancomycin	256 [256]	>128 [32]	128 [64]
Clindamycin	2048 [128]	512 [64]	64 [4]
Erythromycin	256 [128]	16 [1]	64 [0.25]
Aztreonam	4 [4]	64 [32]	0.25 [0.063]
Linezolid	1024 [256]	512 [64]	256 [16]
Piperacillin	4 [4]	>128 [32]	2 [0.125]
Fosfomycin	32 [2]	>256 [256]	16 [8]
Pleuromutilin	1024 [256]	256 [4]	64 [0.5]
Colistin	1 [1]	0.5 [0.25]	0.125 [0.031]
Doxycycline	8 [0.5]	0.25 [0.25]	1 [0.25]
Minocycline	16 [1]	0.5 [0.25]	0.5 [0.063]

6 Tryptic digest of dUSCL di-C_9-KKKK-NH_2 and dUSTBβP 3

A)

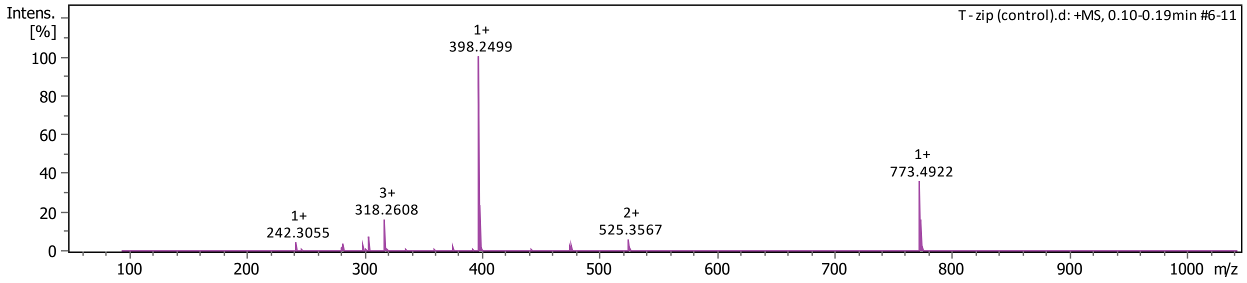

B)

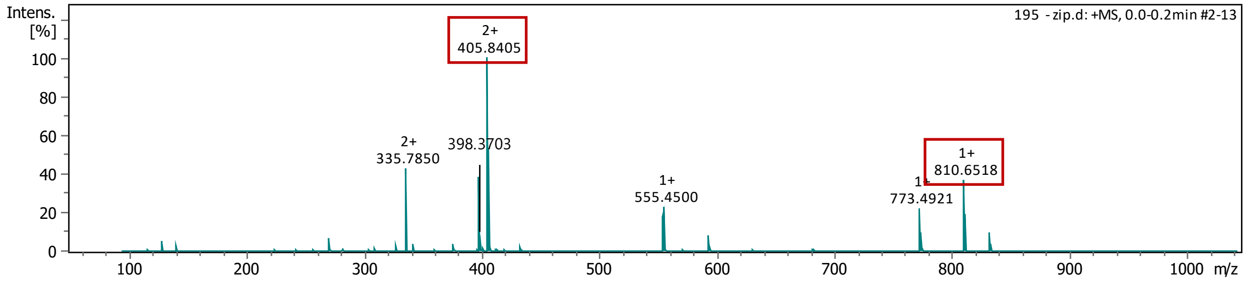

C)

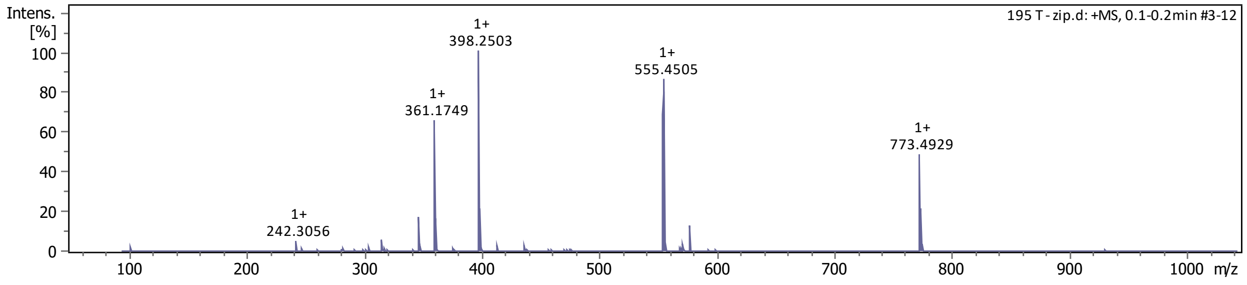

Supplementary Figure 1. Positive-ion ESI-MS spectra of solutions of **(A)** trypsin alone, **(B)** dUSCL di-C_9-KKKK-NH_2 alone, and **(C)** trypsin + dUSCL di-C_9-KKKK-NH_2, in ammonium bicarbonate. Red boxes correspond to singly- and doubly-charged dUSCL di-C_9-KKKK-NH_2.

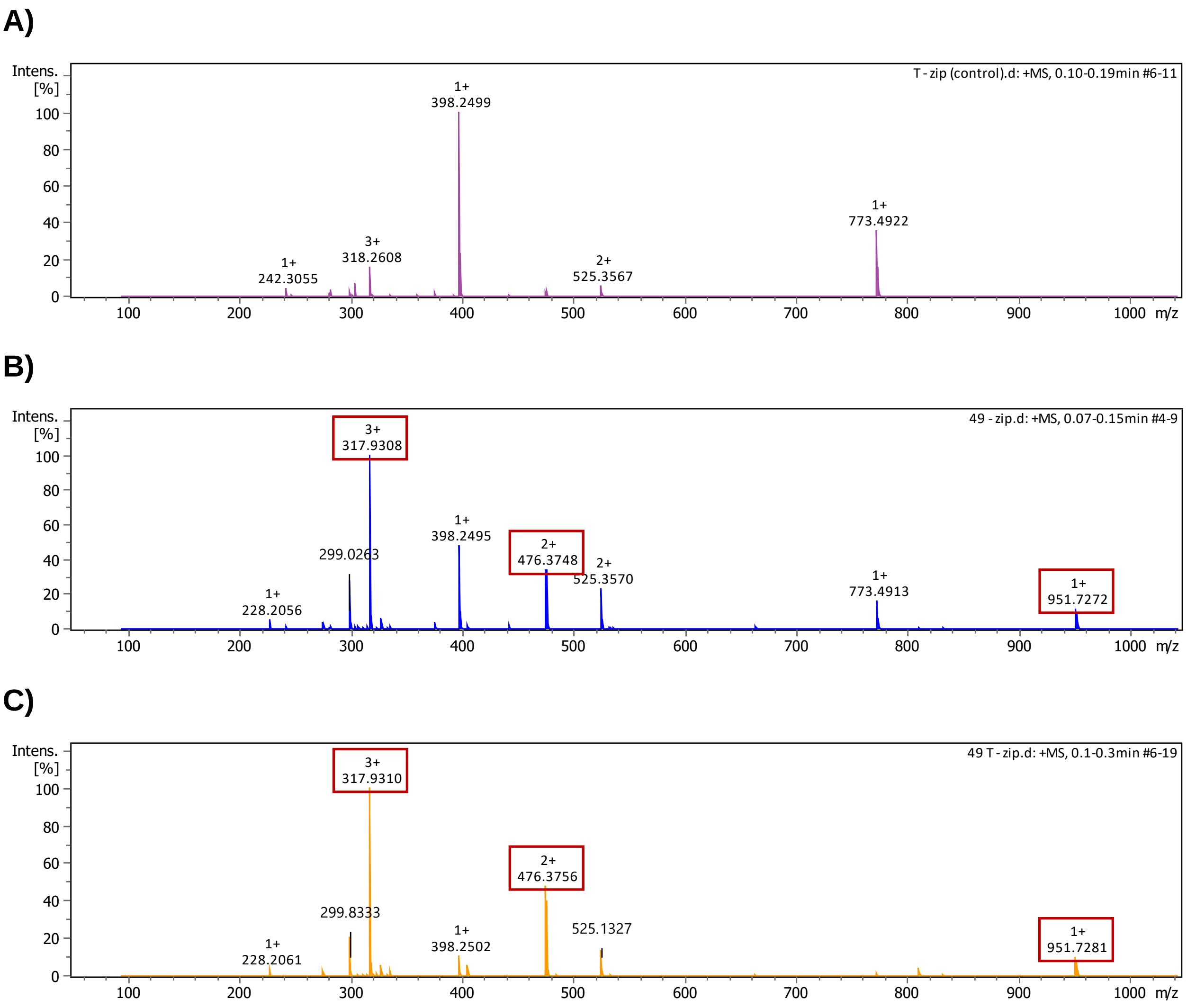

Supplementary Figure 2. Positive-ion ESI-MS spectra of solutions of **(A)** trypsin alone, **(B)** dUSTBβP **3** alone, and **(C)** trypsin + dUSTBβP **3**, in ammonium bicarbonate. Red boxes correspond to singly-, doubly-, and triply-charged dUSTBβP **3**.

7 Cytotoxicity of dUSTBβP 3

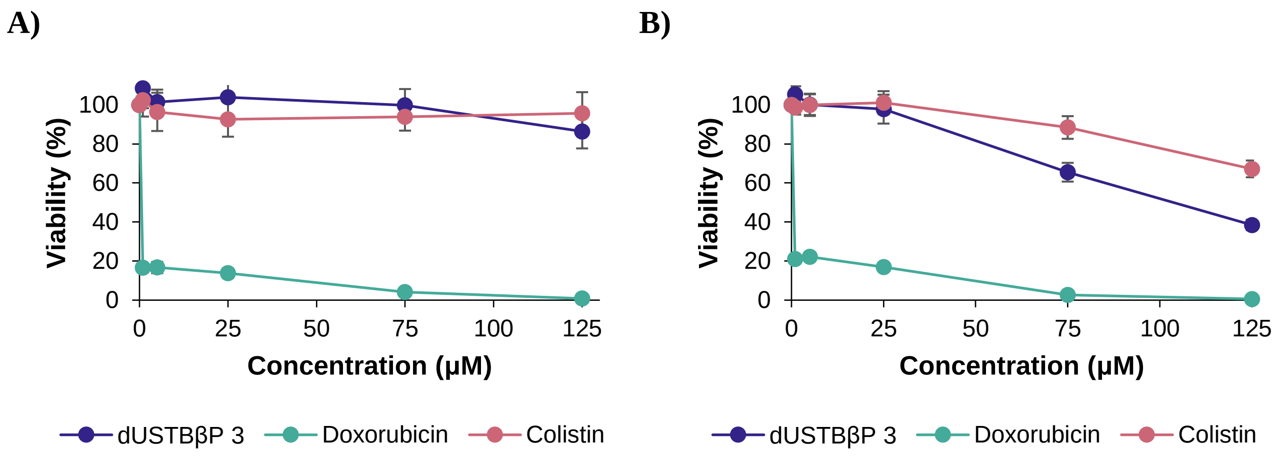

Supplementary Figure 3. Cytotoxicity of dUSTBβP **3** relative to control (vehicle) against **(A)** HEK293 and **(B)** HepG2 cells. Colistin was used as a negative control and doxorubicin was used as a positive control. Results represent the mean ± standard deviation of two independent experiments with five wells for each concentration.

8 NMR spectra of dUSTBβPs

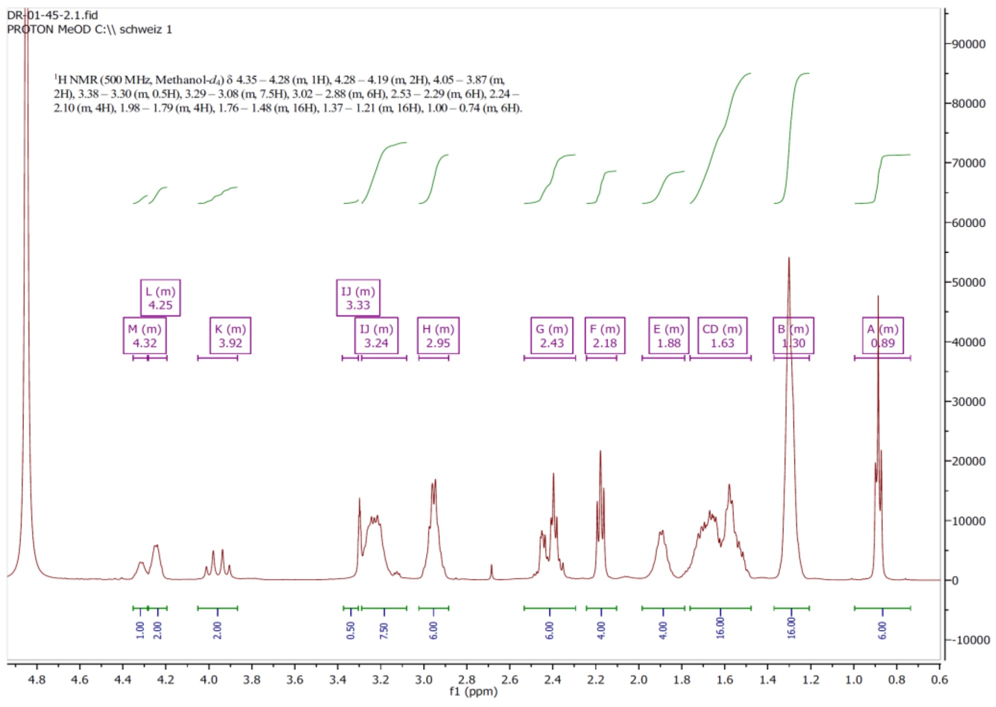

Supplementary Figure 4. ^{1}H spectrum of dUSTBβP **1**.

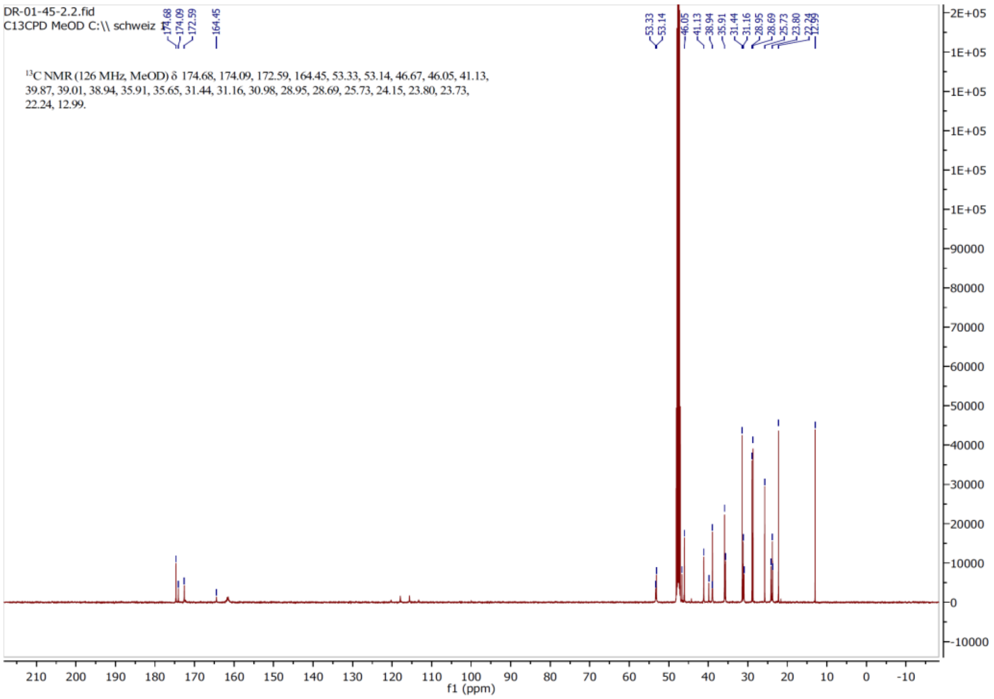

Supplementary Figure 5. ^{13}C spectrum of dUSTBβP **1**.

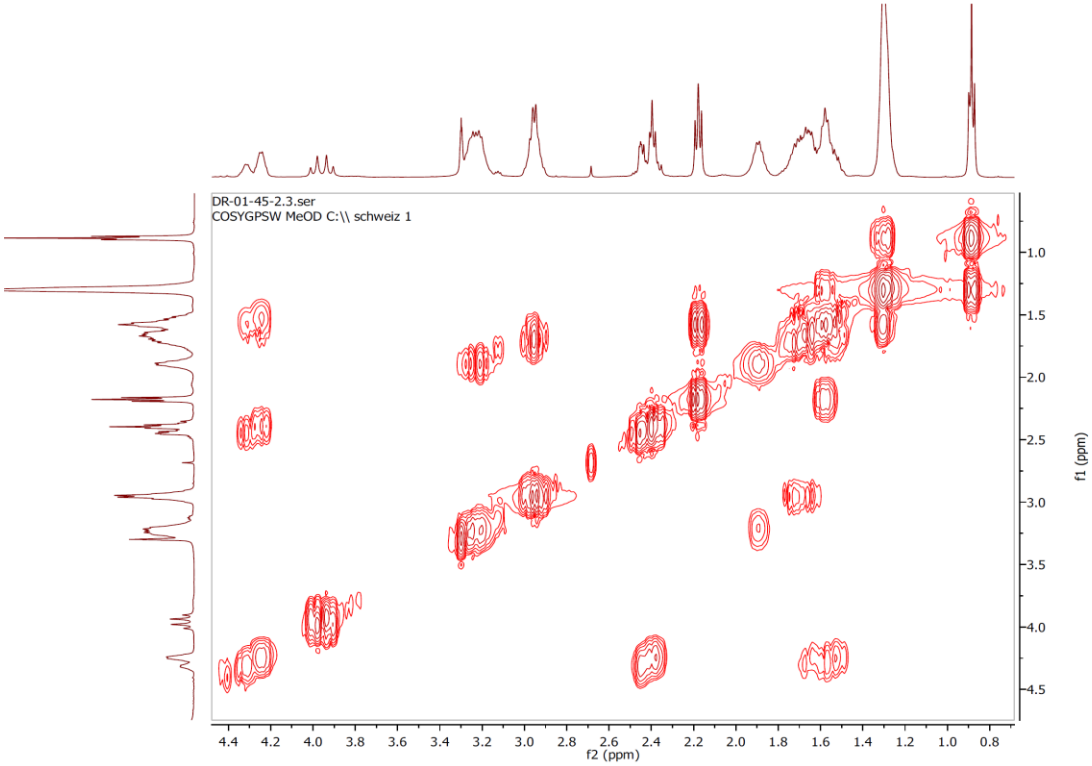

Supplementary Figure 6. COSY spectrum of dUSTBβP **1**.

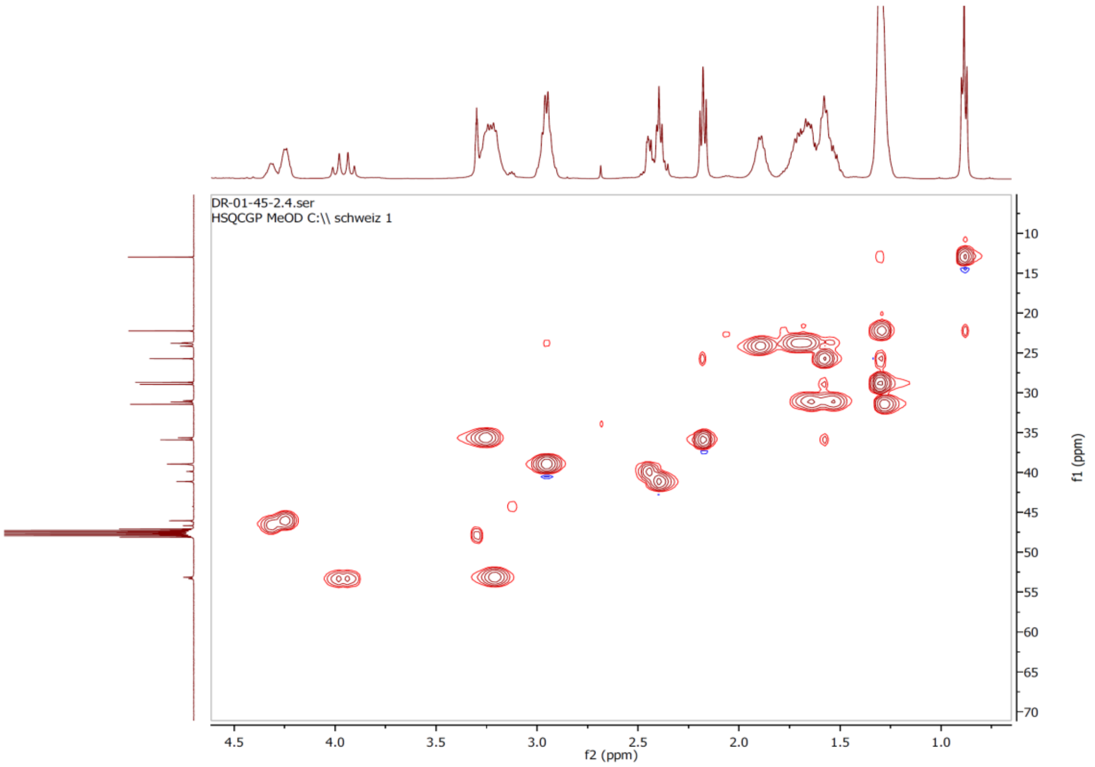

Supplementary Figure 7. HSQC spectrum of dUSTBβP **1**.

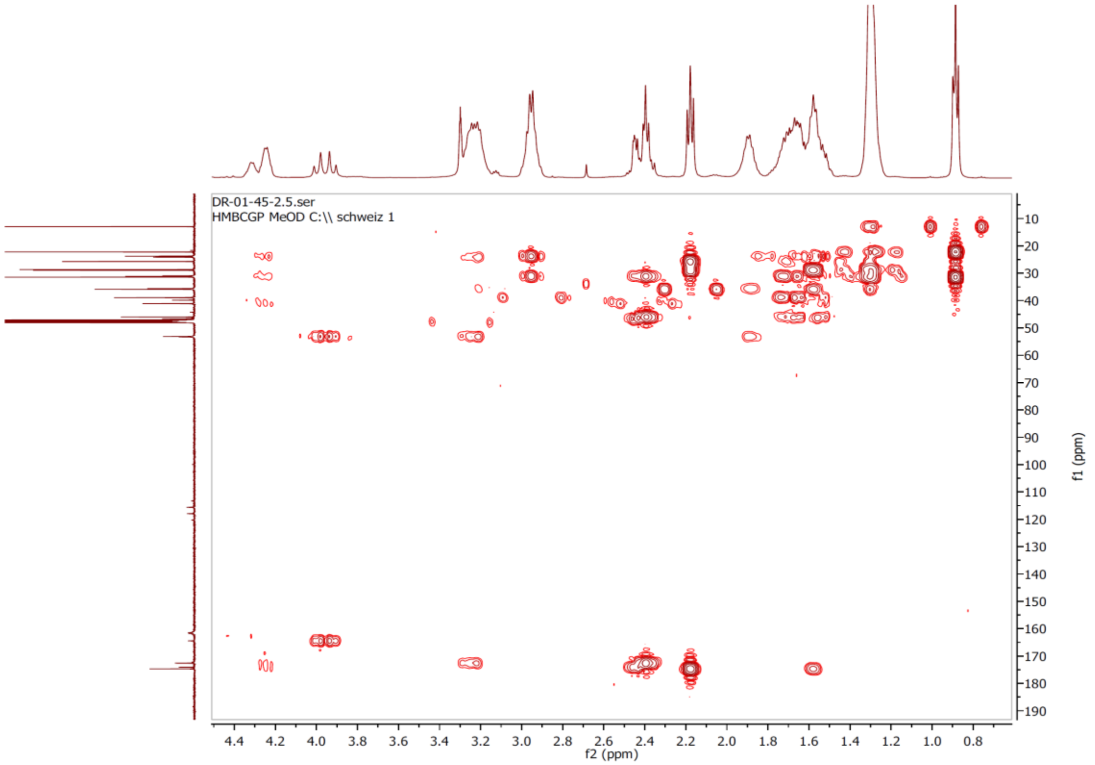

Supplementary Figure 8. HMBC spectrum of dUSTBβP **1**.

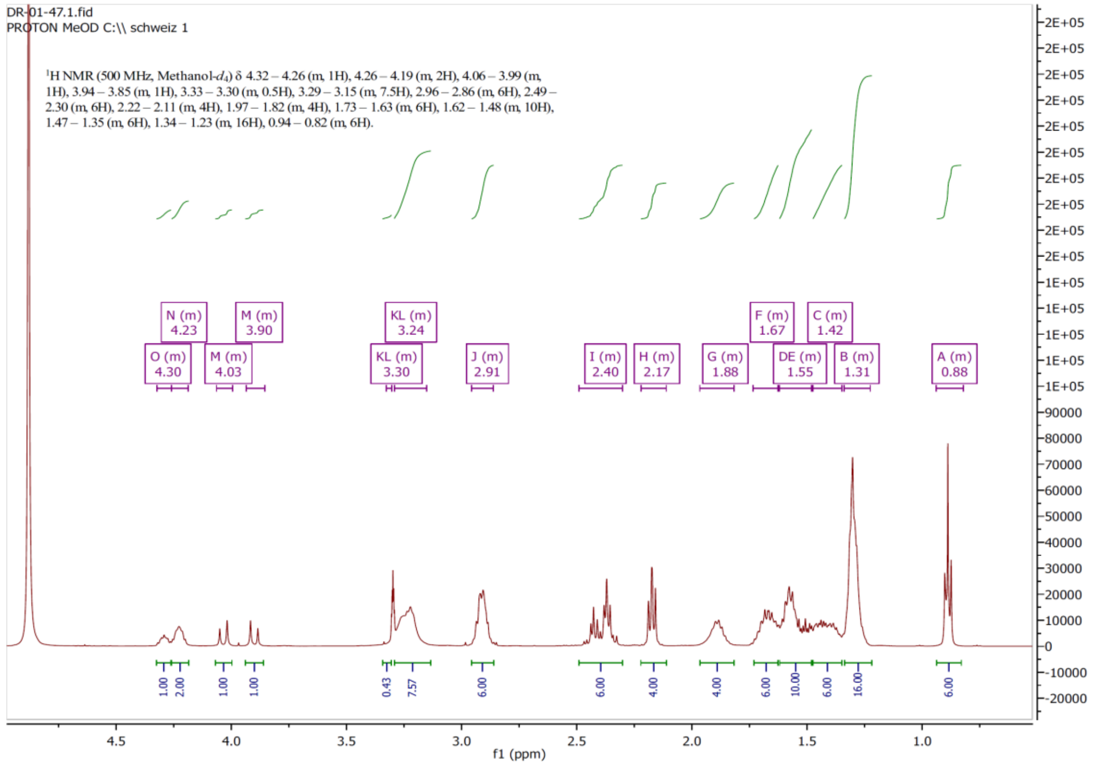

Supplementary Figure 9. ^{1}H spectrum of dUSTBβP **2**.

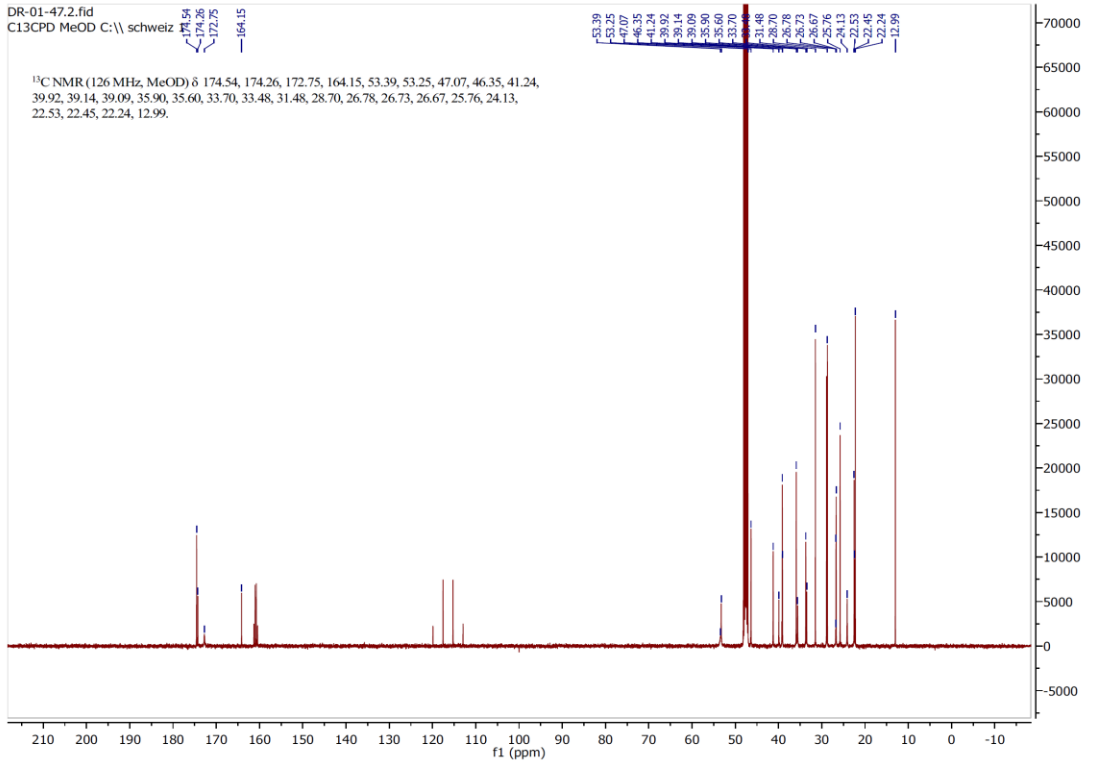

Supplementary Figure 10. ^{13}C spectrum of dUSTBβP **2**.

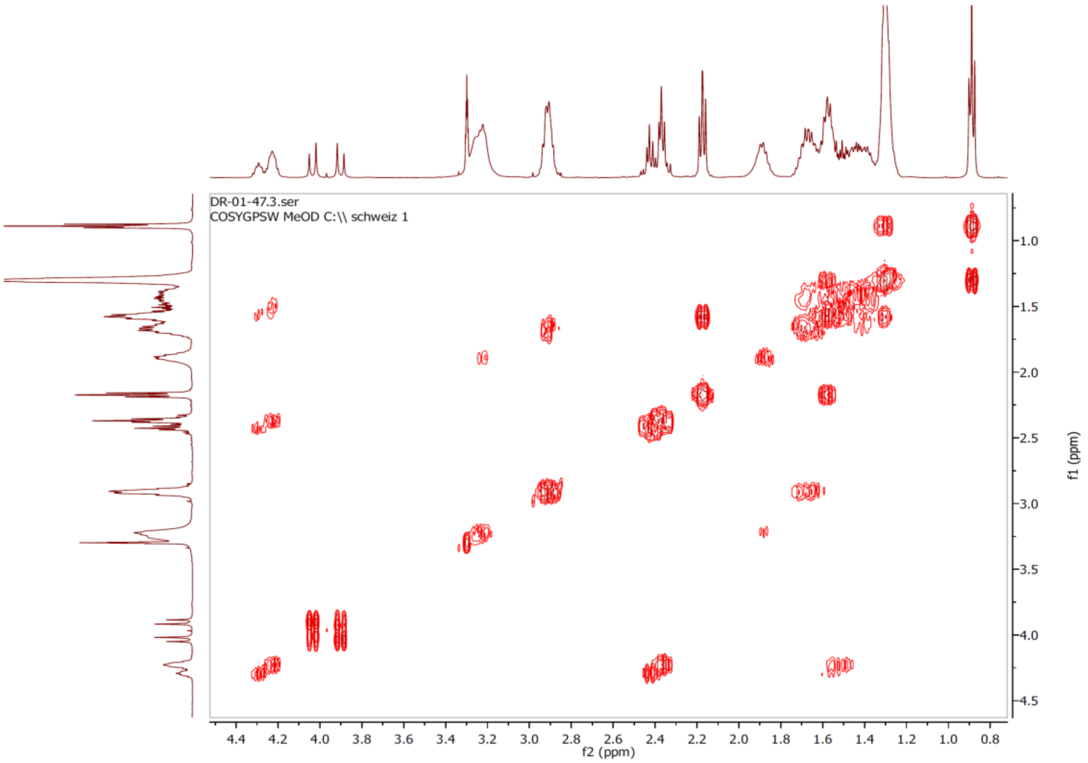

Supplementary Figure 11. COSY spectrum of dUSTBβP **2**.

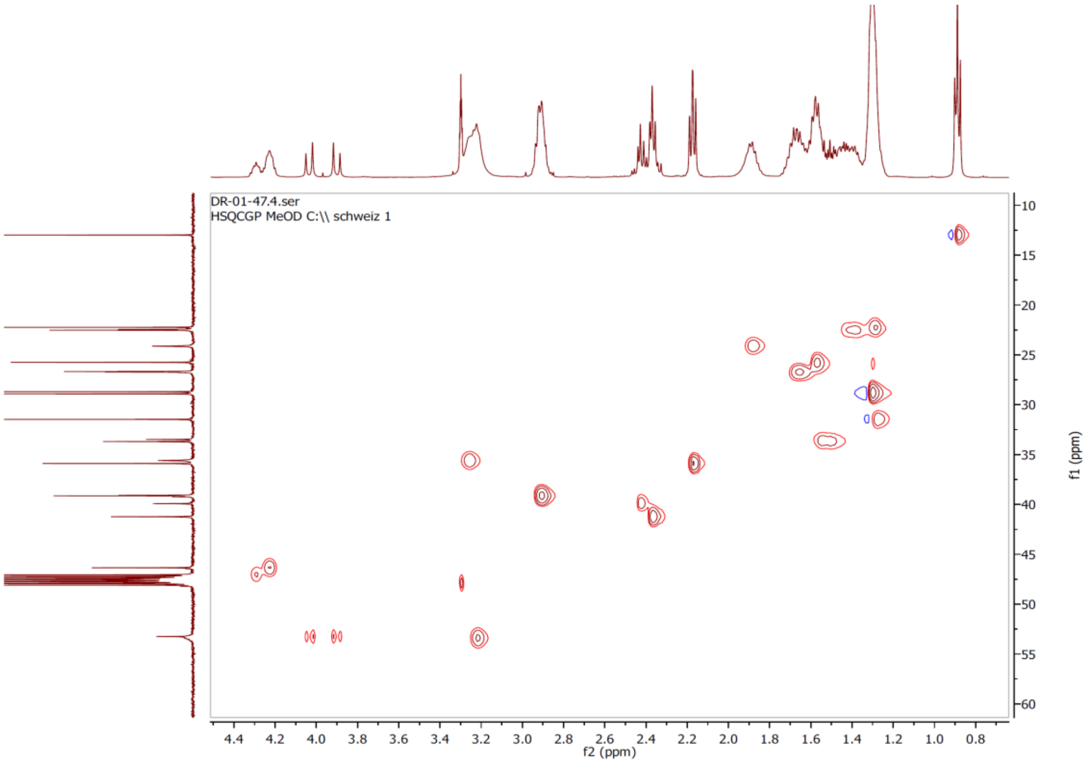

Supplementary Figure 12. HSQC spectrum of dUSTBβP **2**.

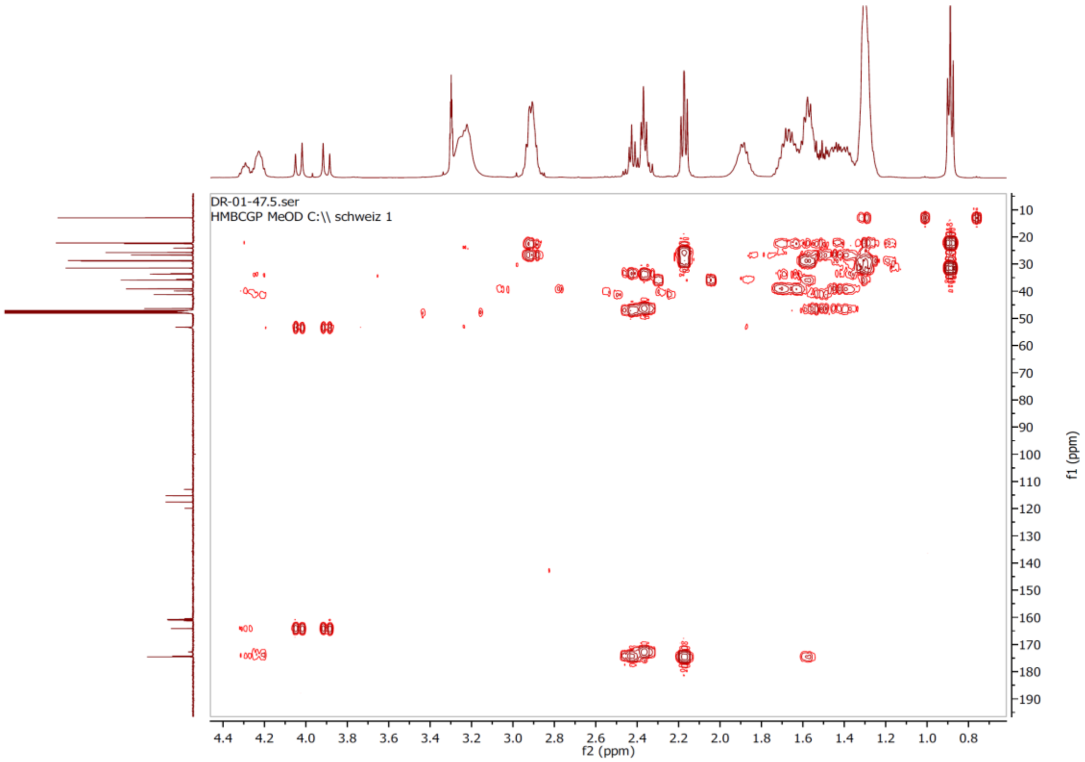

Supplementary Figure 13. HMBC spectrum of dUSTBβP **2**.

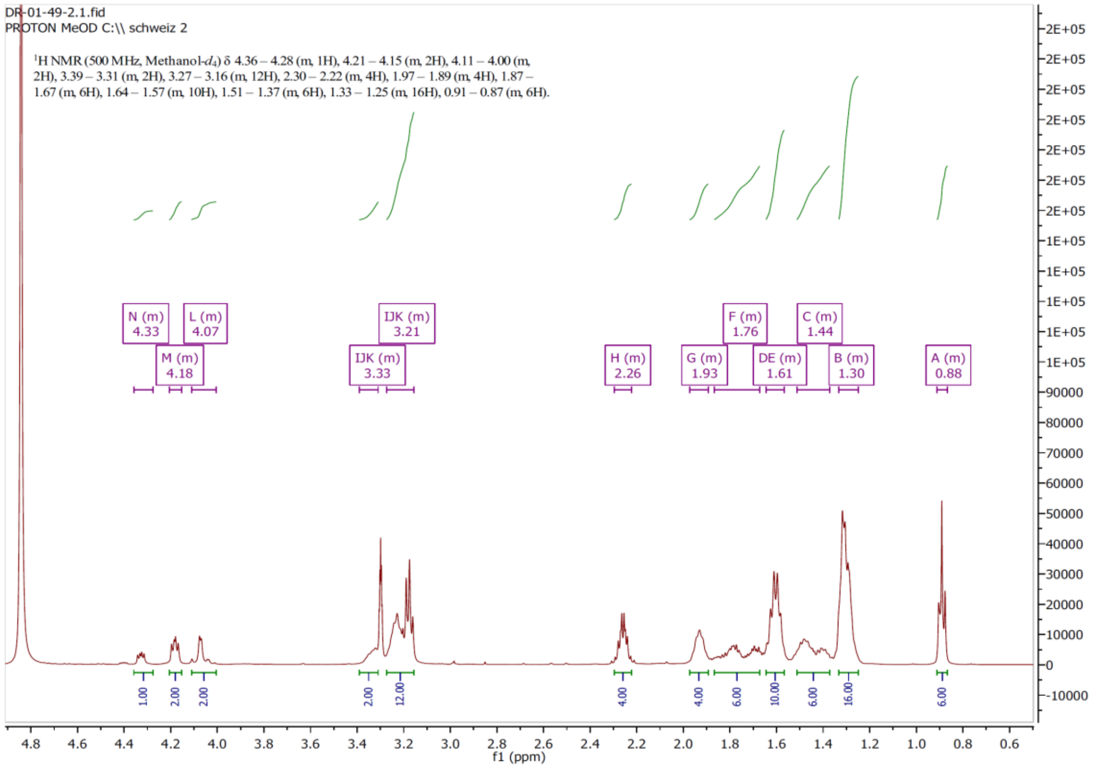

Supplementary Figure 14. ^{1}H spectrum of dUSTBβP **3**.

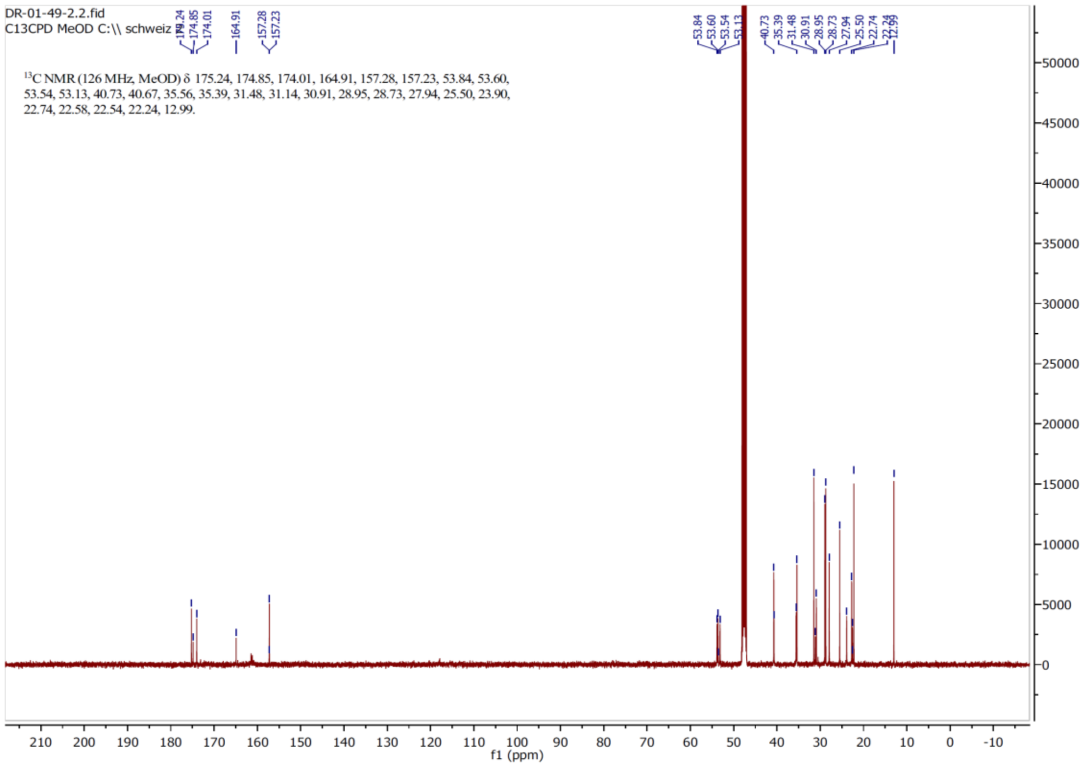

Supplementary Figure 15. ^{13}C spectrum of dUSTBβP **3**.

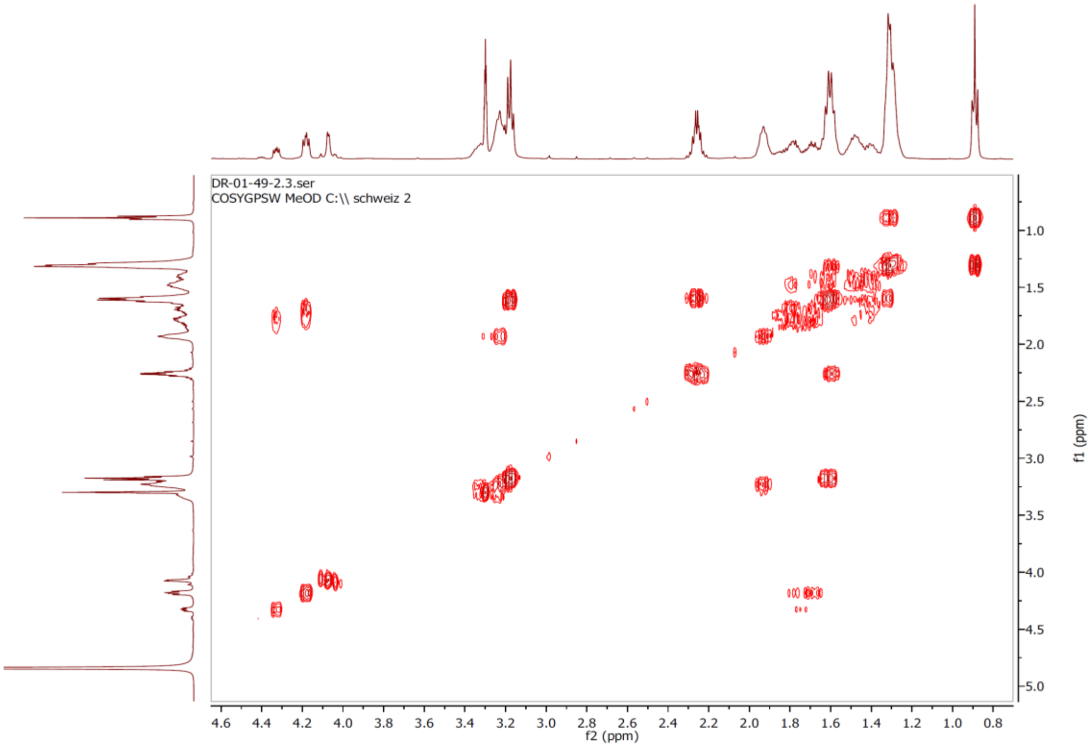

Supplementary Figure 16. COSY spectrum of dUSTBβP **3**.

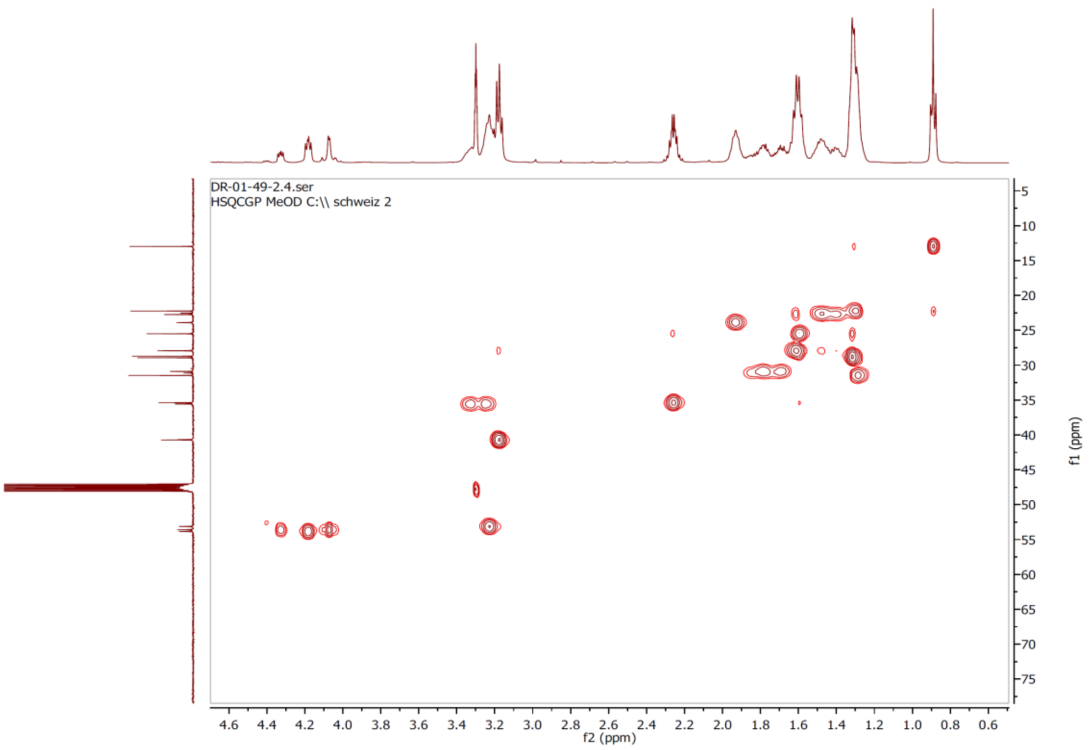

Supplementary Figure 17. HSQC spectrum of dUSTBβP **3**.

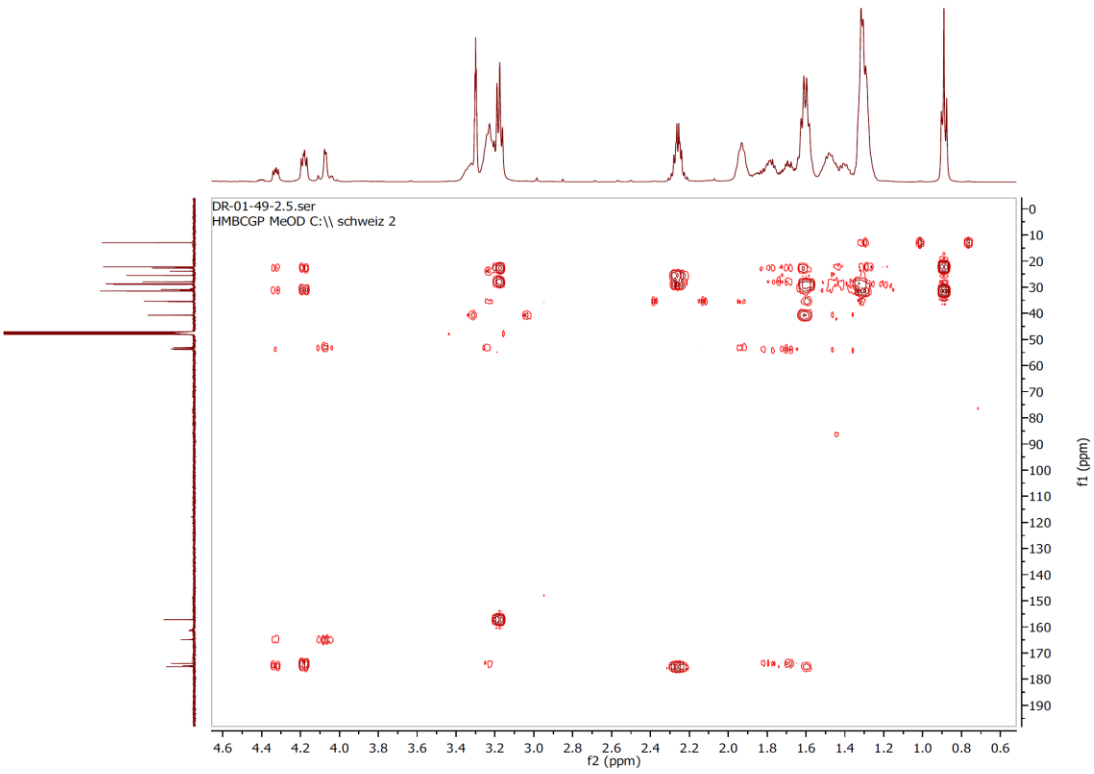

Supplementary Figure 18. HMBC spectrum of dUSTBβP **3**.

9 HPLC chromatograms of dUSTBβPs

Supplementary Table 8. HPLC gradient used for purity analysis.

Time (min)	% Eluent A	% Eluent B
0	100	0
3	90	10
6	40	60
6.5	40	60
7.5	100	0
8	100	0

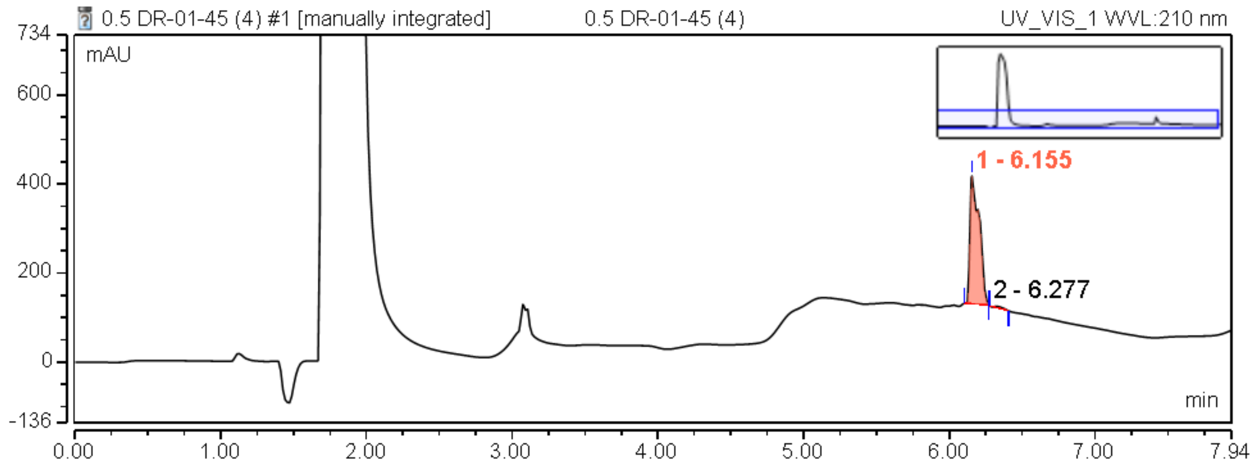

Peak #	Retention Time (min)	Relative Area (%)	Area (mAU*min)	Height (mAU)
1	6.155	98.83	21.0208	286.63
2	6.277	1.17	0.2493	0.00

Supplementary Figure 19. HPLC analysis chromatogram of dUSTBβP **1**. Peak at 1.5 mins corresponds to DMSO.

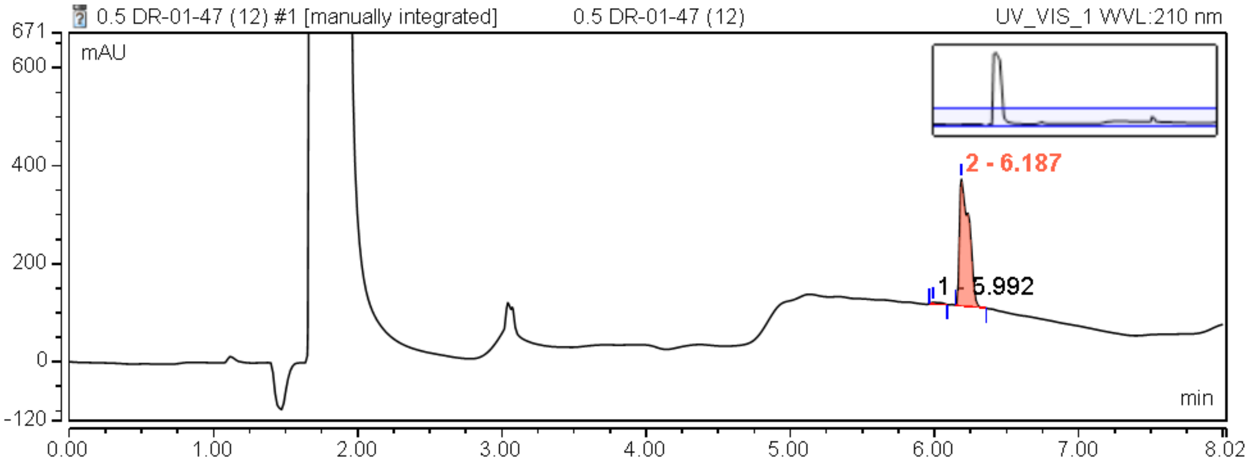

Peak #	Retention Time (min)	Relative Area (%)	Area (mAU*min)	Height (mAU)
1	5.992	1.64	0.3192	3.94
2	6.187	98.36	19.1769	258.33

Supplementary Figure 20. HPLC analysis chromatogram of dUSTBβP **2**. Peak at 1.5 mins corresponds to DMSO.

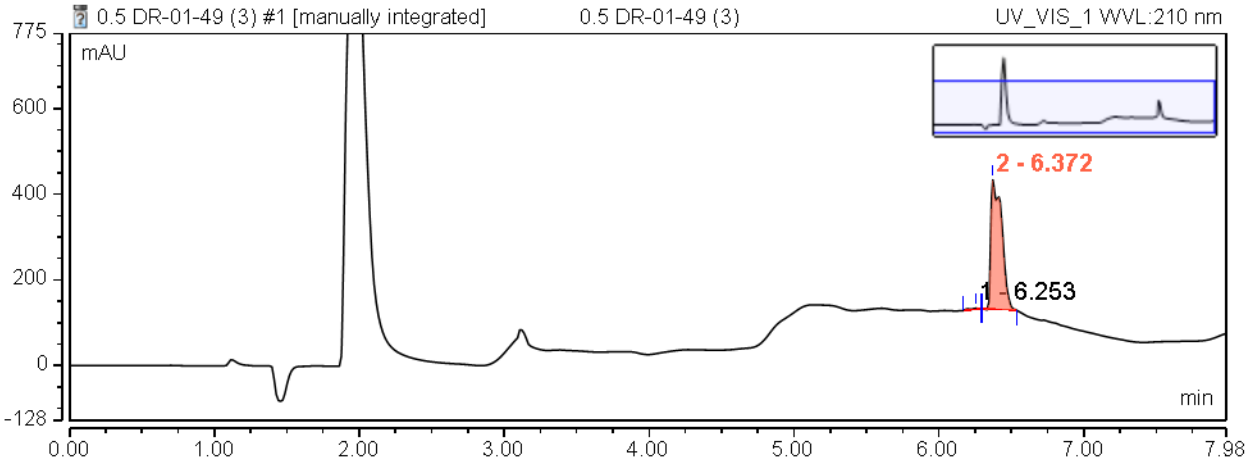

Peak #	Retention Time (min)	Relative Area (%)	Area (mAU*min)	Height (mAU)
1	6.253	0.86	0.2243	2.77
2	6.372	99.14	25.7705	301.53

Supplementary Figure 21. HPLC analysis chromatogram of dUSTBβP **3**. Peak at 1.5 mins corresponds to DMSO.

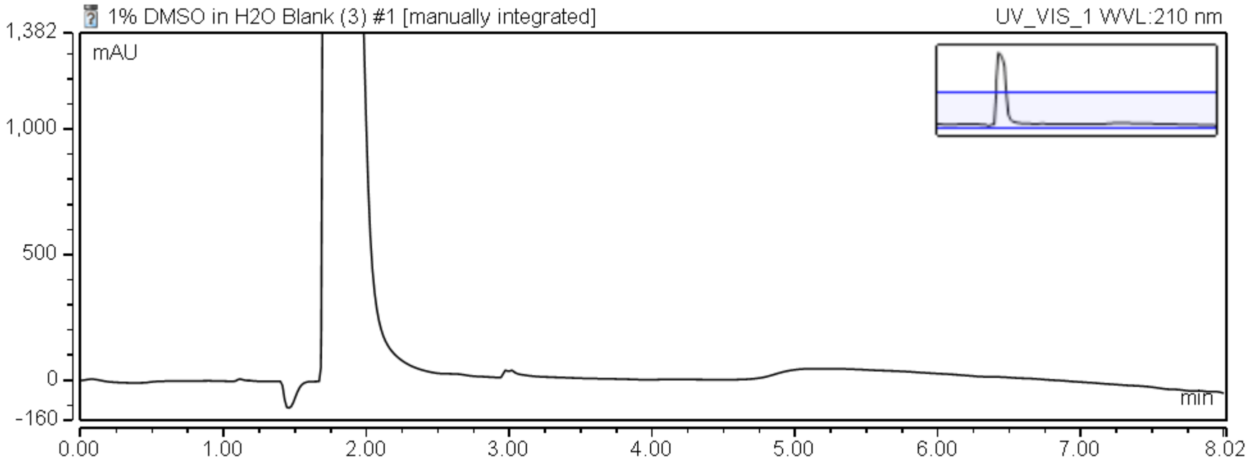

Supplementary Figure 22. HPLC analysis chromatogram of blank (1% DMSO in water).

10 Resistance phenotype of MDR clinical isolates

Supplementary Table 9. MICs (in μg/mL) of various antibiotics against MDR clinical isolates used in the study.

P. aeruginosa	PTZ	A/C	AZT	FOX	CFZ	CTR	CPM	CTX	CAZ	IMI	MER	DOR	ETP	CIP	LEV	MOX	TOB	GEN	AMK	TGC	MIN	DOX	ERC	OMC	CST	CAM
259-96196	64	>32	32	>32	>128	>64	>64	2048	512	32	1024	>1024	>32	>16	256	>16	256	>32	>64	32	32	32	8	64	1	1024
262-101856	64	>32	32	>32	>128	64	32	128	16	32	32	16	>32	>16	64	>16	1024	>32	>64	32	64	1024	8	64	1	2048
264-104354	256	>32	64	>32	>128	>64	32	2048	128	32	64	16	>32	>16	64	>16	128	>32	8	32	32	64	8	64	1	4096
91433	64	>32	512	>32	>128	>64	16	1024	1024	32	16	16	>32	2	ND	16	16	32	>32	32	16	32	16	128	4	8
114228	ND	ND	32	ND	ND	ND	ND	128	8	ND	8	8	ND	ND	ND	ND	2	ND	ND	ND	32	16	8	16	4	ND

A. baumannii	PTZ	FOX	CFZ	CPM	CTX	CAZ	C/T	IMI	MER	CIP	LEV	MOX	TOB	GEN	AMK	TGC	MIN	DOX	ERC	OMC	CST	CAM
AB027	512	ND	>128	>128	>256	ND	>16	32	16	>16	8	8	ND	32	>64	4	0.25	ND	0.5	1	0.25	128
AB031	4	ND	>128	4	16	ND	>16	0.25	1	0.25	0.25	0.12	ND	<0.5	2	8	0.25	ND	0.25	2	0.25	128
LAC-4	ND	ND	ND	ND	I	>16	8	<1	<1	>4	2	ND	>4	>4	4	<4	4	<4	0.06	1	0.125	32
92247	<1	32	128	4	ND	ND	2	ND	4	≤0.06	ND	ND	ND	ND	<1	0.25	0.125	ND	ND	ND	4	ND
110193	ND	ND	ND	ND	ND	ND	ND	ND	ND	≤1	≤1	≤1	ND	ND	ND	ND	1	ND	ND	ND	0.5	128

E. coli	PTZ	A/C	AZT	FOX	CFZ	CPM	CAZ	C/T	IMI	MER	ETP	CIP	LEV	MOX	TOB	GEN	AMK	TGC	MIN	DOX	ERC	OMC	CST	CAM
94393 (mcr-1 +)	≤1	4	≤0.12	4	1	≤0.25	≤0.25	0.25	0.25	≤0.03	≤0.03	0.5	1	1	≤0.5	≤0.5	2	0.25	2	4	0.5	4	4	4
94474 (mcr-1 +)	16	>32	≤0.12	16	4	≤0.25	0.5	0.5	0.25	≤0.03	≤0.03	>16	32	16	32	16	2	1	64	>32	1	16	16	4
107115	>512	>32	>64	>32	>128	>64	>32	>16	8	32	>32	>16	32	16	8	>32	2	0.25	32	>32	0.125	4	0.125	512

K. pneumoniae	PTZ	A/C	AZT	FOX	CFZ	CPM	CAZ	C/T	IMI	MER	ETP	CIP	LEV	MOX	TOB	GEN	AMK	TGC	MIN	DOX	ERC	OMC	CST	CAM
113250	4	4	≤0.12	1	1	1	0.5	2	0.25	≤0.03	≤0.03	≤0.06	0.125	≤0.06	≤0.5	≤0.5	≤1	ND	2	2	1	2	>16	4
113254	<1	2	≤0.12	1	1	1	≤0.2	0.5	0.12	≤0.03	≤0.03	≤0.06	0.0625	≤0.06	≤0.5	≤0.5	≤1	ND	2	128	0.5	4	>16	2
116381	8	16	16	16	>128	16	8	1	0.5	≤0.03	0.12	>16	128	>16	4	≤0.5	≤1	1	64	1	0.5	4	1	>512

E. cloacae	PTZ	A/C	AZT	FOX	CFZ	CPM	CAZ	C/T	IMI	MER	ETP	CIP	MOX	TOB	GEN	AMK	TGC	MIN	DOX	ERC	OMC	CST	CAM
117029	2	16	≤0.12	>32	>128	≤0.25	0.5	0.25	0.25	≤0.03	≤0.03	≤0.06	≤0.06	2	≤0.5	2	0.5	32	>32	0.5	4	0.25	8
118564	2	>32	≤0.12	>32	>128	0.25	0.5	ND	ND	0.12	ND	0.06	0.12	1	1	2	ND	ND	4	ND	ND	>16	ND
121187	1	8	≤0.12	>32	32	0.25	0.5	ND	ND	0.06	ND	0.25	1	32	>32	1	ND	ND	>32	ND	ND	>16	ND

PTZ: piperacillin-tazobactam, A/C: amoxicillin-clavulanic acid, AZT: aztreonam, FOX: cefoxitin, CFZ: cefazolin, CTR: ceftriaxone, CPM: cefepime, CTX: cefotaxime, CAZ: ceftazidime, C/T: ceftolozane-tazobactam, IMI: imipenem, MER: meropenem, DOR: doripenem, ETP: ertapenem, CIP: ciprofloxacin, LEV: levofloxacin, MOX: moxifloxacin, TOB: tobramycin, GEN: gentamicin, AMK: amikacin, TGC: tigecycline, MIN: minocycline, DOX: doxycycline, ERC: eravacycline, OMC: omadacycline, CST: colistin, CAM: chloramphenicol, ND: not determined.
